# Large scale identification of pellicle and cell-free liquid phase associated proteins in *Bacillus amyloliquefaciens* L-17

**DOI:** 10.1016/j.crmicr.2025.100387

**Published:** 2025-04-08

**Authors:** Tassadit Ouidir, Julie Hardouin, Claire-Emmanuelle Marcato-Romain, Elisabeth Girbal-Neuhauser, Yassine Nait Chabane

**Affiliations:** a Laboratoire de Biotechnologies Agroalimentaire et Environnementale (LBAE) URU 4565, Université de Toulouse, IUT de Toulouse Auch Castres, IUT A Paul Sabatier, 24 rue d′Embaquès, Auch 32000, France; b Beaulieu-Lavacant General and Technological Agricultural Education High School, Route de Tarbes, Auch 32020 CEDEX 9, France; c Université de Rouen Normandie, INSA Rouen Normandie, CNRS, Normandie Universite, PBS UMR 6270, Rouen, France; d University of Rouen Normandy, INSERM US 51, CNRS UAR 2026, HeRacLeS PISSARO, Rouen, France

**Keywords:** Pellicle proteins, Cell-free liquid phase, EPS, Proteomic, Antimicrobial peptide, Enzymes

## Abstract

*Bacillus amyloliquefaciens* is a soil-associated and plant growth-promoting bacterium. It is the focus of numerous studies due to its ability to sporulate, form biofilms, produce antimicrobial peptides and commercial enzymes. The ability of *B. amyloliquefaciens*l-17 to form floating biofilm at the air-liquid interface “pellicle” was previously demonstrated. This pellicle exhibits a highly structured architecture which is provided by loosely and tightly matrix bound polysaccharides and proteins. In this study, a first large scale proteomic investigation of both the pellicle and the cell-free liquid phase of l-17 strain was performed. An approach based on physical and chemical extraction of the pellicular matrix combined with protein analysis by mass spectrometry identified 87 weakly matrix-bound proteins and 62 tightly bound proteins. A total of 131 pellicle-associated proteins were identified, including (i) the conserved proteins TasA and TapA, involved in biofilm formation and cohesion (ii) BslA, important for biofilm hydrophobicity (iii) several enzymes that make nutrients available and protect the biofilm from competitors (iv) flagellin and (v) proteins involved in the sporulation process. Proteomic characterization of the cell-free liquid phase underlying the analyzed pellicle allowed the identification of 423 proteins including 118 proteins yet identified in the matrix of the pellicle. The proteins identified specifically in the liquid phase include enzymes involved in the biosynthesis process of non-ribosomal peptides and a variety of commercial enzymes such as proteases, lipases, aminotransferases, peroxidases and phytases. This provides valuable clues to promote the industrial and agricultural application of the cell-free liquid phase of *B. amyloliquefaciens*l-17.

## Highlights

• First Proteomic Mapping of B. amyloliquefaciens l-17 pellicle and liquid phase.
• Physical and chemical EPS extraction and MS analysis reveal loosely or tightly proteins bound to the matrix.
• Pellicle Protein Diversity: roles in Biofilm formation, protection, nutrient acquisition and sporulation.
• Liquid phase specific proteins: commercial enzymes and those of non-ribosomal antimicrobial peptides biosynthesis processes.

## Graphical abstract

**Figure:**
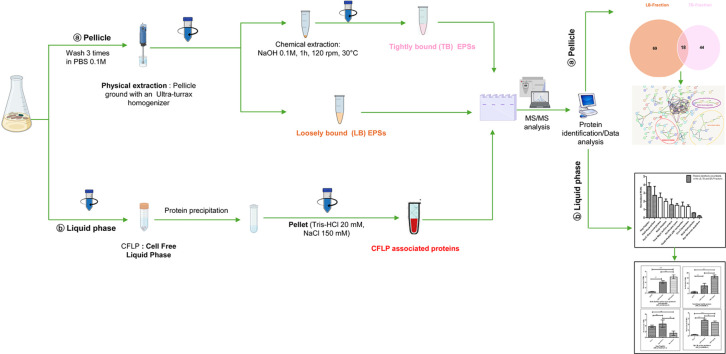

## 1 Introduction

Most bacteria privilege the biofilm lifestyle where they are held together by self-produced extra polymeric substances (EPSs) commonly comprised of polysaccharides, proteins, lipids and DNA (Devi et al., 2023; Flemming and Wuertz, 2019; Karygianni et al., 2020). Biofilm is a highly orchestrated social life strategy where inhabitants engage in coordinated living to fight against the environmental fluctuations (Costerton et al., 1995; Parrilli et al., 2022; Tallawi et al., 2017). The diversity of microorganisms forming biofilms and EPSs composition heterogeneity, provide biofilm different properties which can negatively or positively impact various aspects of our daily life (Devi et al., 2023; Moore-Ott et al., 2022; Ouidir et al., 2022; Pradeep and Arratia, 2022). The beneficial biofilm research field is fast moving owing to the biological relevance in natural systems and human applications including bioremediation, water treatment, food field and agricultural settings (Morikawa, 2006; Mukhi and Vishwanathan, 2022; Philipp et al., 2024).

The non-pathogenic bacterium, *Bacillus subtilis*, has been the major model organism for the study of Gram-positive bacteria for many decades, due to its ease of genetic manipulation and its extensive industrial and agricultural application (Arbour et al., 2023; Arnaouteli et al., 2021; Vlamakis et al., 2013). These studies commonly used the ancestral strain, *B. subtilis* NCIB 3610, in three experimental systems including (i) pellicle, a floating biofilm on air-liquid surfaces (ii) rough colony formed on semi-solid agar surfaces, and (iii) biofilm formation on plant roots (Cairns et al., 2014). In all these models, *B. subtilis* leads cooperative life through cell differentiation, labor repartition and EPSs production (López et al., 2009). The EPSs of *B. subtilis* biofilm are majorly composed of polysaccharides, TasA, TapA, and BslA crucial proteins. Together, they create a complex interaction network that gives the biofilm its architecture and biochemical and physical characteristics (Branda et al., 2006, 2001; Milton and Cavanagh, 2023; Romero et al., 2010). Despite their omnipresence in the natural environments, it is however necessary to note that pellicles are far less studied compared to solid surface attached biofilms (Vaccari et al., 2017). This lifestyle is also found in organisms belonging to the *B. subtilis* species complex, such as members of the *Bacillus amyloliquefaciens* (OGBa) operational group (Ngalimat et al., 2021; Zaidi-Ait Salem et al., 2021b).

*B. amyloliquefaciens* has long been the center of research interest. This ubiquitous species is known for its excellent ability to plant colonization and protection from pathogens (Jiao et al., 2021; Wang et al., 2017). It is considered as plant growth-promoting (PGPR) bacteria due to its abilities to fix nitrogen, solubilize phosphate, and produce siderophores and phytohormones, as well as antimicrobial compounds and a wide range of commercial enzymes (Ngalimat et al., 2021). However, biofilm formed by this species remain very little studied compared to those of *B. subtilis* (Kimani et al., 2016; Zaidi-Ait Salem et al., 2021b; Zhao et al., 2016). Interestingly, Deka et al. (2019) has shown that the EPSs produced by *B. amyloliquefaciens* p16 is able to confer acid tolerance to the bacterium and improve the structural stability of the soil by increasing its aggregation when inoculated. On the other hand, Qiu et al. (2014) identified key proteins involved *in situ* root colonization and biofilm formation in *B. amyloliquefaciens* SQR9 by comparing the proteomic profiles of planktonic and root-colonized SQR9 cells.

During the biofilm or pellicle formation in *Bacillus* species, two populations, sessile and planktonic, coexist and can exchange. Cells from the biofilm can migrate to the planktonic population while cells from the planktonic population can enter the biofilm (El-Khoury et al., 2021). Thus, the cell-free liquid phase (CFLP) seems interesting to explore. Cell-free supernatant of *Bacillus* species, including *B. amyloliquefaciens*, planktonic culture is increasingly attracting researchers because of its richness in bacterial bioactive compounds (Latorre et al., 2016; Naamala et al., 2022; Park et al., 2003; Xu et al., 2019). Several studies have shown its effectiveness for fight against biofilms formed by clinical (Islam et al., 2022; Pedretti et al., 2024) or foodborne pathogens (Hamza et al., 2018; Park et al., 2023), but also against pathogenic fungus attacking plant crops (Gao et al., 2017; Schmiedeknecht et al., 1998; Zhao et al., 2022). This cell-free supernatant is a promising alternative to traditional antimicrobial or anti-biofilm molecules having potential issues related to environmental pollution, microbial resistance and human health. In fact, it is filled with antimicrobial arsenal non-ribosomal peptides (i.e. iturin, surfactin, fengycin) (Puan et al., 2023; Ranjan et al., 2023; Tran et al., 2022). Moreover, the *B. amyloliquefaciens* extracellular medium growth contains various enzymes including α-amylase, proteases, lipases, cellulases, xylanases, pectinases, aminotransferases, barnases, peroxidases, and laccases (Farhat-Khemakhem et al., 2018; Ngalimat et al., 2021).

Results from the whole genome sequence of *B. amyloliquefaciens* l-17 revealed that it could be a potential strain for study of biofilm formation, and for investigation in biocontrol agents against plant pathogens and enzymes production (Zaidi-Ait Salem et al., 2021a). Zaidi-Ait Salem et al. (2021b) underlined the ability of l-17 strain to survive on air-liquid interfaces forming thick floating biofilms. They highlighted that the mature pellicle exhibits rough hydrophobic surface on the air interface and a smooth hydrophilic texture on the surface in contact with the liquid. The same authors reported that the pellicle matrix is composed of loosely and tightly bound polysaccharides and proteins providing the pellicle cohesion and architecture (Zaidi-Ait Salem et al., 2021b). Continuing this work, we have firstly investigated the proteome of the pellicle matrix of *B. amyloliquefaciens* l-17 strain. Therefore, we combined the physical and chemical protocol matrix extraction and the high accuracy tandem mass spectrometry analysis to have the first board view of matrix proteins of L1–7 strain. As far as we know, no proteomic study has been previously carried out on the matrix of mature biofilm or pellicles. In the second part of this present study, we performed, for the first time, a deep proteomic characterization of the CFLP to establish an initial repertoire of interest proteins in *B. amyloliquefaciens* l-17 liquid phase environment.

## 2 Materials and methods

### 2.1 Bacterial strain and growth conditions

*Bacillus amyloliquefaciens*l-17 was provided by the Culture Collection of the Laboratoire de Biotechnologies Agroalimentaire et Environnementale (culture collection WDCM 1016, LBAE-UPS, Auch, France). An overnight culture in rich trypticase soya broth (TSB) medium was performed at 30 °C and shaking at 150 rpm. This pre-culture was used to inoculate a glucose, fructose minimal medium (MMGF) (glucose 10 g⋅*L*^−1^, fructose 10 g⋅*L*^−1^, MgSO_4_ 0.05 g⋅*L*^−1^, C_6_H_11_FeNO_7_(Ferric ammonium citrate) 0.025 g⋅*L*^−1^, H_3_BO_3_ 3 × 10^−5^ g⋅*L*^−1^, CoCl_2_⋅6H_2_O 21 × 10^−5^ g⋅*L*^−1^, ZnSO_4_⋅7H_2_O 11 × 10^−5^ g⋅*L*^−1^, MnCl_2_⋅4H_2_O 4 × 10^−6^ g⋅*L*^−1^, CuSO_4_⋅5H_2_O 2 × 10^−6^ g⋅*L*^−1^, NiCl_2_⋅6H_2_O 1 × 10^−6^ g⋅*L*^−1^, Na_2_MO_4_⋅2H_2_O 3 × 10^−6^ g⋅*L*^−1^, Na_2_HPO_4_ 4.33 g⋅*L*^−1^, K_2_HPO_4_ 2.65 g⋅*L*^−1^, CaCl_2_ 0.0625 g⋅*L*^−1^, NH_4_Cl 6 g⋅*L*^−1^, pH = 7) at final concentration of 10^7^ CFU.mL^−1^. Pellicles in triplicate were formed in 1 L Erlenmeyer flasks containing 400 mL of culture medium for 72 h at 30 °C under static conditions.

### 2.2 EPSs extraction by physical and chemical methods

The extraction of pellicle EPSs is based on the combination of sequenced physical and chemical extractions. As previously described, the pellicle obtained at the air-liquid interface was recovered by forceps and was washed three times with PBS. The underlying liquid phase was used for a parallel proteomic analysis. The pellicle was then suspended in 20 mL of PBS (2 g VSS mL−1) and ground with an ULTRA-TURRAX homogenizer (3 × 15 s and 8000 rpm), aiming to release the loosely bound EPSs (LB-EPSs). After centrifugation of the suspension (20,000 g, 15 min and 4 °C), the supernatant containing the LB-EPSs proteins was recovered and the pellet was resuspended in 20 mL NaOH (0.1 M) to extract tightly bound EPSs (TB-EPSs). After incubation (60 min, 30 °C), the supernatant was separated from the remaining insoluble materials by centrifugation (20,000 g, 15 min and 4 °C) and the obtained soluble extract (TB-EPSs) was recovered. In each LB-EPSs and TB-EPSs fraction, protein concentration was evaluated using the Bradford assay. Then, the LB-EPSs and TB-EPSs were stored at −20 °C for further analysis.

### 2.3 Cell-free liquid phase proteins (CFLP) precipitation

The liquid phase underlying the pellicle was centrifuged (10,000 x g, 20 min, 4 °C) to remove planktonic bacteria. The proteins of this cell-free liquid phase (CFLP) were precipitated with trichloroacetic acid (TCA). One volume of TCA 100 % (w/v) was added to 4 vol of bacteria-free growth medium. After incubation for 15 min at 4 °C, the protein pellet was collected by centrifugation (10,000 x g, 20 min, 4 °C), and washed with ice-cold acetone. Proteins were solubilized in buffer containing Tris–HCl 20 mM, NaCl 150 mM. Protein concentration was evaluated using the Bradford assay, then the sample was stored at −20 °C.

### 2.4 In gel protein digestion

Twenty-five μg of proteins from either the LB-EPSs, TB-EPSs and CFLP fractions were mixed with SDS loading buffer (63 mM Tris–HCl, pH 6.8, 10 mM DTT, 2 % SDS, 0.02 % bromophenol blue, 10 % glycerol), then loaded onto an SDS-PAGE stacking gel (7 %). A short electrophoresis was performed (10 mA, 2 h). After migration, the gels were stained with Coomassie blue and destained with a solution containing 50 % ethanol, 10 % acetic acid and 40 % deionized water. The revealed protein band from each fraction was excised, washed with water, and then immersed in reductive medium (5 mM DTT). Cysteines were irreversibly alkylated with 25 mM iodoacetamide in the dark. Following washing steps in water, gel bands were submitted to protein digestion with trypsin (2 μg per each band). The digestion was achieved after 3 h at 37 °C. Several steps of peptide extraction were performed in H_2_O / CH_3_CN/trifluoroacetic acid (TFA) mixtures (49.5 / 49.5 / 1). The peptide mixtures were dried and stored at −20 °C until MS analysis.

### 2.5 Tandem mass spectrometry

All experiments were done on an LTQ-Orbitrap Elite coupled with an Easy nLC II system (both from Thermo Scientific) and were carried out in three biological replicates. The samples (0.2 µg) were injected onto an enrichment column (C18 PepMap100, Thermo Scientific). The separation was achieved with an analytical column needle (NTCC-360/internal diameter: 100 μm; particle size: 5 μm; length: 153 mm, NikkyoTechnos, Tokyo, Japan). The mobile phase consisted of H_2_O/0.1 % FA (buffer A) and ACN/FA 0.1 % (buffer B). Tryptic peptides were eluted at a flow rate of 300 nL/min using a three-step linear gradient: from 2 to 40 % B over 75 min, from 40 to 80 % B in 4 min and 11 min at 80 % B. The mass spectrometer was operated in positive ionization mode with a capillary voltage and a source temperature set at 1.5 kV and 275 °C, respectively. The samples were analyzed using the CID (collision induced dissociation) method. The first scan (MS spectra) was recorded in the Orbitrap analyzer (*R* = 60,000) with the mass range *m/z* 400–1800. Then, the 20 most intense ions were selected for MS^2^ experiments. Singly charged species were excluded for MS^2^ analysis. Dynamic exclusion of already fragmented precursor ions was applied for 30 s, with a repeat count of 1, a repeat duration of 30 s and an exclusion mass width of ± 10 ppm. Fragmentation occurred in the linear ion trap analyzer with collision energy of 35. All measurements in the Orbitrap analyzer were performed with on-the-fly internal recalibration (lock mass) at *m/z* 445.12002 (polydimethylcyclosiloxane).

### 2.6 Database searches

Raw data files were processed using Proteome Discoverer 1.4 software (Thermo Scientific). Peak lists were searched using the MASCOT search engine (Matrix Science) against *B. amyloliquefaciens* l-17 National Center for Biotechnology Information (NCBI) database (with 4001 entries). Database searches were performed with the following parameters: 2 missed trypsin cleavage sites allowed; variable modifications: carbamidomethylation of cystein, oxidation of methionine. The parent ion and daughter ion tolerances were 5 ppm and 0.35 Da, respectively. For the identification, a false discovery rate below 1 % was selected.

### 2.7 Label-free quantitative and statistical analyses

Label-free quantitative analysis was achieved with spectral counting method in which the abundance of peptide is measured as the number of Peptide Spectral Matching (PSM) events. A protein abundance is measured by the total number of MS/MS spectra associated with it from all its supporting peptides (Lundgren et al., 2010; Ning et al., 2012). Thus, protein abundance was analyzed for the 10 proteins having the highest PSM in each fraction (TB, LB and CFLP). The PSMs were normalized and ANOVA test with significant differences were considered at *p* < 0.05 (Prism 5.0, GraphPad Software, Inc.). The PSMs corresponding to each protein in the different replicates of TB, LB and CFLP samples are provided in the Supplementary information (Table SI).

### 2.8 Subcellular localization, biological function and protein-protein interaction network

Subcellular localization, molecular function and biological process data were acquired after BLAST sequence similarity search for the identified proteins, and from the UniProtKB database (www.uniprot.org). The functional categories of the identified proteins were achieved by Clusters of Orthologous Genes (COGs) database (http://www.ncbi.nlm.nih.gov/COG/)(Galperin et al., 2021) The protein-protein interaction network, including both physical interactions and functional associations (Szklarczyk et al., 2023), was analyzed using STRING (v 12.0) database (https://string-db.org/) with high confidence (0.7) parameter.

## 3 Results and discussion

### 3.1 Identification of pellicle associated proteins

*B. amyloliquefaciens*l-17 bacteria colonizes the air liquid interfaces and coats themselves with exopolymeric substances (EPSs) of their own to form a floating biofilm. Zaidi-Ait Salem et al. (2021b) highlighted the importance of proteins in the aggregation and cohesion of this matrix yet rich in polysaccharide.

**Fig. 1:**
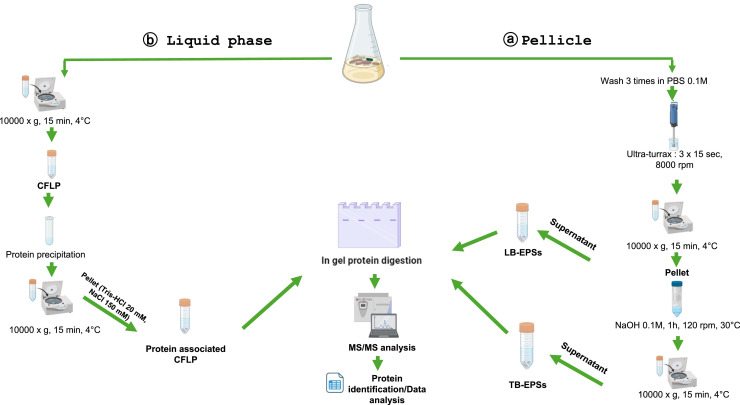
Schematic representation of experimental workflow for analysis of (a) pellicle and (b) liquid phase associated proteins in *B. amyloliquefaciens*l-17. LB Loosely Bound, TB: Tightly Bound, EPSs: Extra-Polymeric Substances, CFLP: Cell Free Liquid Phase.

In this study, we implemented a protocol combining both an ULTRA-TURRAX grinding aiming to release the loosely bound (LB) EPSs and NaOH treatment targeting tightly bound (TB) EPSs. In fact, in biofilms, the LB-EPSs form a loose dispersible slime layer without a distinct boundary. The inner TB-EPSs layer remains tightly packed surrounding the cells (Mahto et al., 2022). Here, we carried out, for the first time, an in-depth proteomic analysis to obtain a global overview of the matrix proteins of this strain. The proteomic strategy is represented in Fig. 1a. After database research, 87 and 62 proteins were identified in LB and TB fractions respectively (Table 1). This result reveals that proteins are distributed in the LB and TB EPS layers and fulfil various functions such as structuring, defense, enzymatic metabolism, etc.

**Table 1:** List of identified proteins in pellicle matrix of *B. amyloliquefaciens*l-17 showing protein distribution in LB and TB fractions and their cellular loacalization and functions.

Accession N^a^	Protein Name^b^	Gene^c^	Molecular function^d^	Biological process^e^	Subcellular localization^f^	% Identity^g^ with *B. subtilis* 168 proteins	COG symbol^h^	LB-fraction^i^	TB-fraction^j^
**Amino acid transport and metabolism**									
WP_003154560.1	aminopeptidase	*ampS*	aminopeptidase activity	proteolysis	cytoplasm	87	E	x	
WP_003154169.1	aspartate-semialdehyde dehydrogenase	*comER*	pyrroline-5-carboxylate reductase activity	L-proline biosynthetic process	unknown	79	E	x	
WP_014305170.1	gamma-glutamyltransferase	*ggt*	proteolysis/glutathione hydrolase activity	leukotriene C4 gamma-glutamyl transferase activity	extracellular region	98	E	x	x
WP_003154048.1	type I glutamate–ammonia ligase	*glnA*	ligase (glu, ATP, nucluotid, Mg, metal binding)	nitrogen catabolite repression of transcription/negative regulation of DNA-templated transcription/cellular response to nitrogen levels	unknown	97	E		x
WP_015388449.1	peptide ABC transporter substrate-binding protein	*oppA*	peptide transmembrane transporter activity	protein transport/establishment of competence for transformation/sporulation	cell membrane	88	E		x
WP_003151992.1	leucyl aminopeptidase	*pepA*	metalloaminopeptidase activity	proteolysis	cytoplasm	76	E	x	
WP_015388480.1	3-phosphoserine/phosphohydroxythreonine transaminase	*serC*	aminotransferase/O-phospho-L‑serine:2-oxoglutarate aminotransferase activity/pyridoxal phosphate binding	amino-acid biosynthesis	cytoplasm	85	EH		x
***Carbohydrate transport and metabolism***									
WP_015388729.1	starch-binding protein	*amyE*	alpha-amylase activity/glycosidase/hydrolase	carbohydrate metabolism	extracellular region	86	G		x
WP_003151102.1	fructose-bisphosphate aldolase	*fbaA*	fructose-bisphosphate aldolase activity/tagatose-bisphosphate aldolase activity	lyase/glycolysis/sporulation	cytoplasm	99	G		x
WP_003151611.1	type I glyceraldehyde-3-phosphate dehydrogenase	*gapA*	Oxidoreductase/NADP binding	glycolysis	unknown	97	G	x	
WP_015388768.1	glycoside hydrolase family 3 protein	*nagZ*	beta-N-acetylhexosaminidase activity	peptidoglycan biosynthetic process	extracellular region/plasma membrane	75	G		x
WP_015387727.1	oxalate decarboxylase family bicupin	*oxdC*	oxalate decarboxylase activity	oxalate metabolic process	periplasmic space/cytoplasm	87	G	x	x
WP_015388558.1	pectate lyase	*pel*	pectate lyase activity/metal ion binding	pectin catabolic process	extracellular region	76	G		x
WP_014306184.1	glycoside hydrolase family 68 protein	*sacB*	unknown	carbohydrate metabolism	unknown	90	G		x
WP_003151103.1	fructose-6-phosphate aldolase	*tal*	aldehyde-lyase activity	carbohydrate metabolic process	cytoplasm	90	G		x
WP_015388167.1	carbohydrate-binding protein	*xynD*	glycosidase/hydrolase	carbohydrate metabolism/polysaccharide degradation/xylan degradation	extracellular region	90	G		x
WP_003150769.1	glycoside hydrolase family 16 protein	*ydhD*	glycosidase/hydrolase/ chitin binding	carbohydrate metabolism/polysaccharide degradation/sporulation	extracellular region/spore wall	72	GE		x
***Cell cycle control, cell division, chromosome partitioning, Inorganic ion transport and metabolism, Cell wall/membrane/envelope biogenesis***									
WP_003155789.1	multicopper oxidase family protein	*cotA*	oxidoreductase	sporulation	periplasmic space	77	D	x	
WP_015387430.1	spore coat protein	*cotF*	unknown	sporulation	spore coat	71	D	x	x
WP_015388201.1	N-acetylmuramoyl-l-alanine amidase	*cwlC*	N-acetylmuramoyl-l-alanine amidase activity/peptidoglycan binding/hydrolase	peptidoglycan catabolic process/sporulation	extracellular region/spore wall	77	D	x	x
WP_003153438.1	stage IV sporulation protein A	*spoIVA*	ATP hydrolysis activity	sporulation	spore wall/endospore cortex	96	D	x	
WP_003155477.1	gamma-type small acid-soluble spore protein	*sspE*	unknown	sporulation	unknown	84	D	x	
WP_003155170.1	hypothetical protein	*yrpD*	unknown	unknown	unknown	88	D	x	
***Cell wall/membrane/envelope biogenesis***									
WP_015388018.1	alanine racemase	*alr2*	catalyzes the interconversion of l-alanine and d-alanine. pyridoxal phosphate binding	D-alanine biosynthetic process/ peptidoglycan biosynthetic process	cytoplasm	84	M	x	
WP_015387749.1	chitosanase	*csn*	glycosidase/hydrolase	carbohydrate metabolic process	extracellular region	88	M		x
WP_003154166.1	4‑hydroxy-tetrahydrodipicolinate synthase	*dapA*	4‑hydroxy-tetrahydrodipicolinate synthase activity	mino-acid biosynthesis	cytoplasm	94	ME	x	
WP_003155677.1	LTA synthase family protein	*ltaS1*	metal ion binding	cell wall organization	extracellular region/plasma membrane	85	M		x
WP_032865795.1	glycoside hydrolase family 18 protein	*sleL*	glycosidase Hydrolase	sporulation	spore coat	78	M		x
WP_015388671.1	hypothetical protein	*Unknown*	unknown	unknown	extracellular region	74	M	x	
WP_003151206.1	endo-1,4-beta-xylanase XynA	*xynA*	glycosidase/Hydrolase	carbohydrate metabolism/polysaccharide degradation/xylan degradation	unknown	95	MG	x	
WP_015388168.1	glucuronoxylanase xynC	*xynC*	glycosidase/Hydrolase	carbohydrate metabolism/xylan degradation/polysaccharide degradation/ sphingolipid metabolic process	extracellular region	98	M	x	
WP_015388752.1	DUF1906 domain-containing protein	*ybfG*	unknown	unknown	cell membrane	84	M		x
WP_003154742.1	glycoside hydrolase family 18 protein	*ydhD*	chitin binding chitin activity	chitin catabolic process/carbohydrate metabolism/sporulation	extracellular region	72	M		x
WP_003150840.1	choloylglycine hydrolase family protein	*yxeI*	hydrolase	antibiotic resistance	unknown	76	M		x
WP_003150773.1	DUF3298 and DUF4163 domain-containing protein	*yxiT*	unknown	unknown	unknown	84	M		x
WP_088005523.1	PQQ-binding-like beta-propeller repeat protein	*yxaL*	increases the processivity of the PcrA helicase but does not bind to DNA		extracellular region	76	M	x	
***Defence mechanisms***									
WP_004393188.1	peroxiredoxin	*ahpC*	antioxydant/oxidoreductase/peroxidase/stress response	hydrogen peroxide catabolic process/response to oxidative stress	cytoplasm	98	V	x	x
WP_007614902.1	alkyl hydroperoxide reductase subunit F	*ahpF*	oxidoreductase/translocase	electron transport	cell membrane	95	V		x
WP_015388172.1	cellulase family glycosylhydrolase	*ahpF*	oxidoreductase/translocase	electron transport	cell membrane	95	V		x
WP_015387507.1	catalase	*katE*	catalase activity/heme binding	hydrogen peroxide catabolic process/response to oxidative stress	cytoplasm	84	V		x
WP_139,886,654.1	class A beta-lactamase	*penP*	hydrolase	antibiotic resistance	unknown	91	V		x
WP_152,514,787.1	multifunctional 2′,3′-cyclic-nucleotide 2′-phosphodiesterase/3′-nucleotidase/5′-nucleotidase	*yfkN*	metal ion binding/nucleotide binding	nucleotide catabolic process	extracellular region/ periplasmic space	74	VF	x	
***Energy production and conversion***									
WP_003151173.1	F0F1 ATP synthase subunit beta	*atpD*	unknown	unknown	cell membrane	96	C	x	
WP_003151167.1	F0F1 ATP synthase subunit C	*atpE*	proton-transporting ATP synthase activity, rotational mechanism/ lipid binding	ATP synthesis/Hydrogen /ion transport	plasma membrane/multi-pass membrane protein	100	C		x
WP_003152455.1	malate dehydrogenase	*mdh*	oxidoreductase	tricarboxylic acid cycle	cytoplasm	96	C		x
WP_015388516.1	FAD-binding oxidoreductase	*ygaK*	oxidoreductase activity/ FAD binding	unknown	Unknown	85	C	x	
WP_003156110.1	FAD-binding oxidoreductase	*yvdP*	oxidoreductase	sporulation	spore coat	98	C	x	
WP_003151173.1	F0F1 ATP synthase subunit beta	*atpD*	unknown	unknown	cell membrane	96	C	x	
***Inorganic ion transport and metabolism***									
WP_003155700.1	spore coat protein CotJC	*cotJC*	spore coat composition	sporulation	Unknown	96	P	x	
WP_003156778.1	ABC transporter substrate-binding protein	*feuA*	part of the ABC transporter complex FeuABC/YusV involved in import of the catecholate siderophores bacillibactin and enterobactin.	iron ion transport	Cytoplasm/ cell membrane	83	P	x	
WP_003155420.1	catalase KatA	*katA*	oxidoreductase/peroxidase	hydrogen peroxide catabolic process	cytoplasm	94	P	x	x
WP_012118382.1	methionine ABC transporter substrate-binding lipoprotein MetQ	*metQ*	unknown	Amino-acid transport	cell membrane	87	P	x	x
WP_003153772.1	superoxide dismutase	*sodF*	superoxide dismutase activity		unknown	80	P		x
WP_014305921.1	right-handed parallel beta-helix repeat-containing protein	*ywoF*	unknown	unknown	extracellular region	74	P		x
WP_003156656.1	metal ABC transporter substrate-binding protein	*znuA*	metal ion binding	zinc ion transport/cell adhesion	plasma membrane	83	P	x	
***Lipid transport and metabolism***									
WP_003154310.1	acyl carrier protein	*acpA*	growing fatty acid chain in fatty acid biosynthesis	fatty acid metabolism	cytoplasm	100	I	x	
WP_003156672.1	triacylglycerol lipase	*estA*	triglyceride lipase activity	lipid catabolic process	extracellular region	76	I	x	
WP_003156315.1	glucose 1-dehydrogenase	*gdh*	oxidoreductase/glucose 1-dehydrogenase [NAD(P)] activity	sporulation	unknown	90	I	x	
WP_003155182.1	glucose 1-dehydrogenase	*yhxC*	oxidoreductase	unknown	unknown	92	I	x	
***Nucleotide transport and metabolism/Inorganic ion transport and metabolism***									
WP_003150717.1	IMP dehydrogenase	*guaB*	oxidoreductase	GMP biosynthesis/purine biosynthesis	cytoplasm	96	F	x	
WP_015387718.1	assimilatory sulfite reductase (NADPH) flavoprotein subunit	*cysJ*	flavin adenine dinucleotide binding/FMN binding	cysteine biosynthetic process/amino-acid biosynthesis	cytoplasm	78	FP	x	
***Post translational modification, protein turnover, chaperones***									
WP_003155195.1	S8 family serine peptidase	*aprE*	serine protease	fibrinolysis/proteolysis/sporulation	extracellular region	85	O	x	
WP_003154079.1	S8 family peptidase	*aprX*	serine-type endopeptidase activity	proteolysis	extracellular region/cytoplasm	83	O		x
WP_015388265.1	S8 family serine peptidase	*bpr*	serine-type endopeptidase activity	proteolysis	extracellular region	70	O	x	x
WP_014305002.1	HslU–HslV peptidase ATPase subunit	*clpY*	ATP hydrolysis activity	proteolysis	unknown	93	O	x	
WP_004264608.1	ATP-dependent zinc metalloprotease FtsH	*ftsH*	ATP-dependent peptidase activity	protein catabolic process/cell cycle/sporulation	plasma membrane	96	O	x	x
WP_014304883.1	S8 family peptidase	*isp*	serine-type endopeptidase activity	proteolysis	unknown	93	O	x	
WP_003155497.1	serine protease	*mpr*	serine-type endopeptidase activity	proteolysis	extracellular region	74	O		x
WP_003151600.1	hypothetical protein	*nprB*	metal ion binding	proteolysis	extracellular region	83	O		x
WP_015388283.1	M4 family metallopeptidase	*nprE*	metalloendopeptidase activity	proteolysis	extracellular region	83	O		x
WP_003155249.1	peptidylprolyl isomerase	*prsA*	protein folding AND TRANSPORT	peptidyl-prolyl cis-trans isomerase activity	plasma membrane	85	O	x	
WP_014306056.1	S8 family serine peptidase	*vpr*	serine-type endopeptidase activity	proteolysis	extracellular region	80	O	x	x
WP_007410388.1	ATP-dependent protease ATP-binding subunit ClpC	*clpC*	ATP hydrolysis activity/chaperone/repressor	chaperone/establishment of competence for transformation	cytoplasm	98	O	x	
***Replication, recombination and repair***									
WP_003153447.1	non-specific DNA-binding protein Hbs	*hbs*	DNA binding	chromosome condensation	cytoplasm	100	L	x	
***Secondary metabolites biosynthesis, transport and catabolism***									
WP_003151745.1	aldo/keto reductase	*yvgN*	aldo-keto reductase (NADP) activity	small molecule metabolic process	unknown	90	Q		x
***Signal transduction mechanisms***									
WP_003153224.1	transporter substrate-binding domain-containing protein	*artP*	ligand-gated monoatomic ion channel activity	amino acid transport	plasma membrane	82	TE		x
WP_003154064.1	RNA chaperone Hfq	*hfq*	RNA binding	translation	cytoplasm	98	T	x	x
WP_015388734.1	TerD family protein	*yceC*		Stress response	cytoplasm	82	T		x
WP_003156648.1	TerD family protein	*yceD*	stress response	Unknown	unknown	93	T	x	
***Transcription***									
WP_003156143.1	cold shock protein CspC	*cspC*	nucleic acid binding	transcription/transcription regulation	cytoplasm	98	K		x
***Translation, ribosomal structure and biogenesis***									
WP_004264654.1	50S ribosomal protein L25/general stress protein Ctc	*ctc*	5S rRNA binding	translation	cytoplasm	71	J	x	
WP_007410399.1	elongation factor G	*fusA*	translation elongation factor activity	protein biosynthesis	cytoplasm	98	J	x	
WP_003155605.1	type I methionyl aminopeptidase	*mapB*	initiator methionyl aminopeptidase activity	proteolysis	cytoplasm	94	J		x
WP_004393063.1	50S ribosomal protein L9	*rplI*	structural constituent of ribosome/ ARN binding	translation	cytoplasm	91	J	x	
WP_003156433.1	50S ribosomal protein L10	*rplJ*	structural constituent of ribosome/ ARN binding	translation	cytoplasm	95	J	x	
WP_003156436.1	50S ribosomal protein L7/L12	*rplL*	structural constituent of ribosome/ ARN binding	translation	cytoplasm	100	J	x	
WP_003154288.1	50S ribosomal protein L19	*rplS*	structural constituent of ribosome/ ARN binding	translation	cytoplasm	91	J	x	x
WP_003152495.1	50S ribosomal protein L20	*rplT*	structural constituent of ribosome/ ARN binding	translation	cytoplasm	99	J	x	
WP_003152662.1	50S ribosomal protein L27	*rpmA*	structural constituent of ribosome/ ARN binding	translation	cytoplasm	98	J		x
WP_003156464.1	30S ribosomal protein S10	*rpsJ*	structural constituent of ribosome/ ARN binding	translation	cytoplasm	100	J	x	
WP_003154296.1	30S ribosomal protein S16	*RPSP*	structural constituent of ribosome/ ARN binding	translation	cytoplasm	98	J		x
WP_003156451.1	elongation factor Tu	*tuf*	translation elongation factor activity	translation	cytoplasm	97	J	x	
WP_015387848.1	SGNH/GDSL hydrolase family protein	*rpmE2*	structural constituent of ribosome/ ARN binding	translation	cytoplasm	91	J		x
WP_003156431.1	50S ribosomal protein L1	*rplA*	structural constituent of ribosome/ ARN binding	translation	cytoplasm	91	J	x	
***Unknown***									
WP_003154023.1	lytic polysaccharide monooxygenase	*ctc*	Unknown/Ribonucleoprotein	unknown/translation	unknown/cytoplasm	50	Unknown	x	
WP_003155052.1	peptide-binding protein	*appA*	peptide transmembrane transporter activity	peptide transport/sporulation	periplasmic space	73	Unknown	x	
WP_003152178.1	DUF1016 domain-containing protein/biofilm surface layer hydrophobin BslA	*bslA/yuaB*	biofilm formation	unknown/ Biofilm formation	extracellular region	74	Unknown	x	x
WP_003150988.1	hypothetical protein	*bslB*	surface hydrophobicity/minor role in biofilm architecture	Unknown /Biofilm formation	extracellular region	79	Unknown	x	x
WP_015388185.1	hypothetical protein	*cotC*	Unknown	Unknown/sporulation	unknown	45	Unknown	x	x
WP_003154111.1	outer spore coat protein CotE	*cotE*	identical protein binding	sporulation	spore coat	96	Unknown	x	x
WP_021734090.1	hypothetical protein	*cotG*	unknown	sporulation	Unknown	81	Unknown	x	
WP_015388659.1	sporulation hydrolase CotR	*cotR*	hydrolase activity	lipid degradation/ sporulation	spore coat	96	Unknown		x
WP_015388113.1	peptoglycan endopeptase	*cwlS*	lytic endotransglycosylase activity	cell wall biogenesis/degradation/proteolysis	cell surface	68	Unknown		x
WP_015388651.1	hypothetical protein	*floT*	unknown	unknown/regulation of cell shape	unknown	28	Unknown	x	
WP_015387631.1	flagellin	*hag*	unknown/structural molecule activity	bacterial-type flagellum	unknown/extracellular region	56	Unknown	x	
WP_003151908.1	hypothetical protein	*mpr*	unknown/serine‑type endopeptidase activity	unknown/proteolysis	unknown/extracellular region	31	Unknown	x	
WP_015387670.1	carboxylesterase/lipase family protein	*pnbA*	Unknown/cholinesterase activity	unknown	unknown	63	Unknown	x	x
WP_003154135.1	stage V sporulation protein SpoVS	*spoVS*	nucleic acid binding	sporulation /cell division	unknown	100	Unknown	x	
WP_003155280.1	alpha/beta-type small acid-soluble spore protein	*sspB*	double-stranded DNA binding	DNA topological change/sporulation	unknown	89	Unknown		x
WP_003154036.1	hypothetical protein	*ssuD*	unknown/alkanesulfonate monooxygenase activity	unknown/alkanesulfonate catabolic process	unknown	30	Unknown	x	
WP_015388007.1	amyloid fiber anchoring/assembly protein TapA	*tapA*	unknown/required for the proper anchoring and polymerization of TasA amyloid fibers at the cell surface		extracellular region/bacterial biofilm matrix	47	Unknown		x
WP_015388008.1	SipW-dependent-type signal peptide-containing protein/biofilm matrix protein TasA	*tasA*	major protein component of the biofilm extracellular matrix /amyloid fibers that bind cells together in the biofilm/antibacterial/spore coat assembly	sporulation	extracellular region/Forespore intermembrane space	84	Unknown	x	x
WP_015388356.1	phage tail sheath family protein	*xkdK*	unknown	unknown	unknown	86	Unknown		x
WP_015388720.1	GTP-binding protein	*yciC*	GTP binding/hydrolase activity		unknown	84	Unknown	x	
WP_015387422.1	DUF1259 domain-containing protein	*ycxD*	unknown/transaminase activity	unknown/alpha-amino acid metabolic process	unknown	27	Unknown	x	x
WP_014305702.1	hydrolase	*yddQ*	unknown/hydrolase activity	unknown	unknown	31	Unknown		x
WP_024084955.1	YdhK family protein	*ydhK*	unknown	unknown	unknown	61	Unknown		x
WP_015388281.1	hypothetical protein	*ylaE*			unknown	52	Unknown		x
WP_014305020.1	insulinase family protein	*ymfH*	metallopeptidase activity	proteolysis	unknown	86	Unknown		x
WP_015388142.1	IseA DL-endopeptidase inhibitor family protein	*yoeB*			unknown	77	Unknown	x	
WP_014304250.1	DUF4879 domain-containing protein	*yolA*			unknown	27	Unknown	x	
WP_003153543.1	hypothetical protein	*yqgA*	unknown	unknown	unknown/extracellular region	52	Unknown	x	
WP_003152143.1	type 1 glutamine amidotransferase	*yraA*	unknown/peptidase activity	unknown/proteolysis	unknown	38	Unknown		x
WP_015387776.1	YukJ family protein	*yukJ*	unknown	unknown	unknown	81	Unknown		x
WP_015387655.1	ribonuclease	*yvaE*	unknown/amino-acid betaine transmembrane transporter activity	unknown/transport	unknown/plasma membrane	43	Unknown	x	
WP_015387689.1	M28 family peptidase	*ywaD*	unknown/peptidase activity	unknown/proteolysis	unknown/extracellular region	48	Unknown		x
WP_003151188.1	VWA domain-containing protein	*ywmC*	unknown	unknown	unknown	66	Unknown		x
WP_187,650,984.1	hypothetical protein	*ywmD*	unknown	unknown	unknown	21	Unknown		x
WP_152,514,778.1	hypothetical protein	*YycH*	unknown/metal ion binding	unknown	unknown/ plasma membrane	25	Unknown		x
WP_003151740.1	hypothetical protein		unknown	unknown	unknown	81	Unknown	x	
WP_003155828.1	DUF1906 domain-containing protein		unknown	unknown	unknown	49	Unknown	x	
WP_004393267.1	hypothetical protein		unknown	unknown	unknown	66	Unknown	x	
Total									72

a Accession number

b Protein name in the NCBI database for *B. amyloliquefaciens*l-17.

c Gene designation.

d Subcellular localization.

e molecular function and.

f biological process data obtained from the UniProKB database after BLAST sequence similarity searches.

g Identity ( %) in UniProt Protein BLAST: percentage of identical amino acids between the *B. amyloliquefaciens*l-17 proteins sequence and that of *B. subtilis* 168.

h COG category symbole: functional categories according Clusters of Orthologous Genes (COG) database.

i LB-fraction: proteins loosly bound to the matrice that were detached by mechanical disruption of the pellicle.

j TB-fraction: proteins tightly bound to the matrice that were chemically extracted of the pellicle. In green, the identified proteins in l-17 strain having low % identity (<70 %) with that of *B. subtilis* 168.

**Fig. 2:**
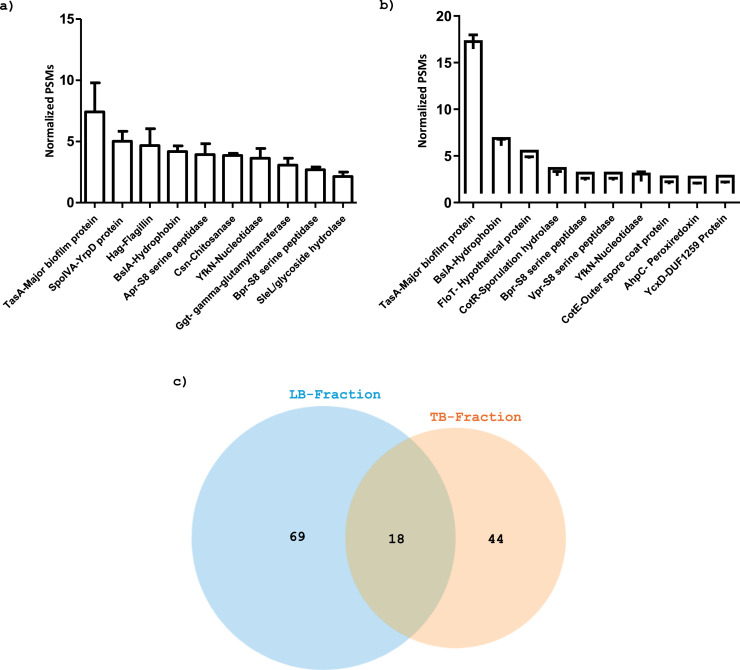
Protein abundance assessment in the (a) loosely bound (LB) and (b) tightly bound (TB) fractions in *B. amyloliquefaciens*l-17 pellicle matrix. Analysis was performed by the spectral counting method and focused on the ten proteins with the higher PSM (Peptide Spectral Match) parameter in each fraction. (c) Venn Diagram showing the distribution of identified proteins in LB and TB fractions. LB fraction contains proteins detached by mechanical disruption of the pellicle. TB fraction includes proteins chemically extracted from the pellicle.

The SipW-dependent-type signal peptide (WP_015388008.1), homologous to *B. subtilis* biofilm matrix protein TasA (84 % sequence identity), is over-presented in both LB and TB fractions (Fig. 2a and b). This result is not surprising since this protein has already been characterized as the major protein component of the *B. subtilis* biofilm matrix, where it has been reported to form functional amyloid fibers contributing to biofilm structure and stability (Böhning et al., 2022). Among the major proteins identified in LB and TB fractions, we also found the DUF1016 domain-containing protein (WP_003152178.1) having 74 % sequence identity with *B. subtilis* 168 biofilm surface layer hydrophobin BslA (Fig. 2a and b). This protein is required for hydrophobicity and architecture of *B. subtilis* biofilms (Arnaouteli et al., 2016). Interestingly, the flagellin (WP_015387631.1), *hag* gene product, is among the major proteins in LB-fraction (Fig. 2a). This can be explained by the fact that some bacteria regain their motility to enter a planktonic lifestyle in the liquid phase. In *B. thuringiensis* 407, Houry et al. (2012), discovered that a subpopulation consisting of 0.1–1 % of cells within a biofilm, referred to as stealth swimmers, could remain motile thanks to their flagella creating transient tunnels highlighting the reversible elastic properties of the matrix. The over-presence of flagellin in the LB-fraction might suggest the presence of stealth swimmers in *B. amyloliquefaciens* l-17 pellicle. This hypothesis could be confirmed using time-lapse confocal laser microscopy at regular intervals to visualize and highlight the involvement of flagella in biofilm structuring or dispersion.

Of the proteins identified, 18 (13 %) were found in both LB and TB fractions (Table 1) and Forty-four proteins (34 %) were specifically identified in the TB-fraction and 69 (53 %) in the LB-fraction (Fig. 2c). This may suggest that the pellicle of *B. amyloliquefaciens* l-17 is more provided in loosely bound proteins that were detached by mechanical disruption of the pellicle. As far as we know, no work using the EPSs sequential extraction to characterize other monospace biofilm models has been reported in the literature. This strategy is often used to study multispecies biofilms such as granular activated sludge (Bou-Sarkis et al., 2024; Caudan et al., 2012) and membrane bioreactors (Lin et al., 2014; Teng et al., 2020). For example, in a membrane bioreactor, Teng et al. (2020) showed that the LB-EPSs had a higher ratio of proteins to polysaccharides than TB-EPSs. Yu et al. (2010) found that LB-EPSs fraction contained much higher proteins than TB-EPSs fraction in aerobic granules. It is widely accepted that sample preparation is an essential stage affecting the overall efficiency of proteomic studies. Prefractionation method allows to visualize a deeper picture of proteome and promoted identification of the less abundant proteins which are buried in the complex mixture (Duong and Lee, 2023). Our results support this, since 131 unique proteins were identified in the pellicle matrix of *B. amyloliquefaciens* l-17 (Table 1) thanks to combining physical and chemical EPSs extraction steps. An additional step extraction, based on a robust urea treatment, would be necessary to access to highly aggregated proteins of amyloid and/or hydrophobic type (Zaidi-Ait Salem et al., 2021b).

We compared our results with the only proteomic study that highlights proteins involved in the first phase of biofilm formation or *in situ* roots colonization in *B. amyloliquefaciens* SQR9 (Qiu et al., 2014). The discussed proteins related to SQR9 strain biofilm formation are not identified in the l-17 strain. This might be explained by the difference in experimental conditions, model biofilms, bacterial strains, and proteomic strategies used in the two studies. We have therefore extended our comparison to other species close to *B. amyloliquefaciens*, such as *B. Subtilis.*

### 3.2 Description of pellicle associated proteins

Subcellular localization, molecular function and biological process data of the identified proteins were obtained from the UniProtKB database (www.uniprot.org) (Table 1). However, we were faced with the lack of directly available data for the proteins of the *B. amyloliquefaciens* l-17 strain. We did BLAST sequence similarity searches for each of the 131 identified proteins *via* Uniprot database. We noticed that most of these proteins (77 %) have a strong homology (up to 70 % sequence identity) with those of *B. subtilis* 168 strain for which the databases are more informed (Table 1). It is known that these two species are genetically very close even though physiologically and biochemically they are clearly distinct (Ngalimat et al., 2021; Welker and Campbell, 1967; Zhang et al., 2016). We therefore focused on this homology to collect data on the cellular location, molecular function, biological process, and protein-protein interaction of identified proteins. For the 29 proteins having low homology (<70 % sequence identity) with that of *B. subtilis* 168, we considered that this information is unknown (Table 1).

#### 3.2.1 Subcellular localization of pellicle associated proteins

**Fig. 3:**
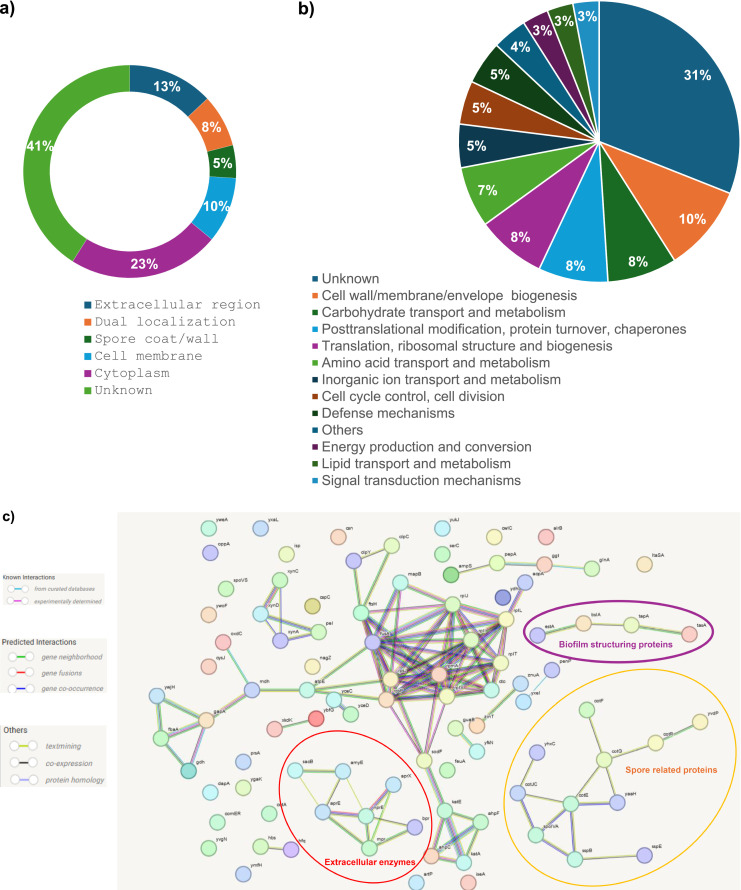
Distribution of proteins identified in *B. amyloliquefaciens*l-17 in the pellicle matrix according to their (a) cellular localization and (b) their functional categories. The cell membrane localization includes plasma membrane, periplasmic space and cell-surface bounded proteins. The category “Others" groups together functional categories with <3 proteins (i.e. transcription; replication, recombination and repair; nucleoid transport and metabolism; secondary metabolites biosynthesis, transport and catabolism). (c) Protein-protein interaction map of the identified pellicle associated proteins in the l-17 strain visualized by STRING program with high confidence parameter. The analysis was achieved using gene names, the corresponding protein names are reported in Table 1.

Among the identified matrix pellicle proteins, 13 % were predicted with an extracellular region localization (Fig. 3a). These include, among others, the major proteins TasA, BslA and flagellin (*hag* gene product). Some proteins (8 %) were predicted with dual localization. For example, the glycoside hydrolase family 3 protein (WP_015388768.1), homologous to the Beta-hexosaminidase NagZ of *B. subtilis* 168 (75 % sequence identity), is predicted with both extracellular and cytoplasmic subcellular locations. This protein is associated with peptidoglycan recycling, for which a crucial role in modulating biofilm accumulation has been demonstrated in *Neisseria gonorrhoeae* (Bhoopalan et al., 2016). Otherwise, we found that the pellicle matrix contains a large fraction of cytoplasmic (23 %) and membrane proteins (10 %) including plasma membrane, periplasmic space and cell-surface bounded proteins. These results implicate the involvement of cell lysis in modulating biofilm proteome composition (Fong and Yildiz, 2015). Several studies reported that *B. subtilis* can sporulate within the biofilm matrix (Azulay et al., 2021; Faille et al., 2014; Srinivasan et al., 2018). The spores are found enriched at the tips of the so-called fruiting bodies which are small visible aerial projections that rise from the surface of a biofilm (Branda et al., 2001). Interestingly, we emphasize that few proteins (5 %) are associated with the coat or wall spore, suggesting that *B. amyloliquefaciens* l-17 could also sporulate inside the pellicle matrix. Microscopic analysis seems to be necessary to confirm this and provide more information on the distribution of spores within the pellicle. We also note that the location of several proteins (41 %) remained unknown.

#### 3.2.2 Biological function of pellicle associated proteins

The 131 identified pellicle associated proteins were classified in clusters of orthologous genes (COGs) categories to have a clearer vision of their physiological role (Table 1). The distribution in various functional categories is summarized in Fig. 3b The protein-protein interaction network mapped by STRING database highlighted three main protein groups which will be discussed, in more depth, in the following sections (Fig. 3c).

#### 3.2.3 Biofilm structuring proteins

As described in Section 3.1, TasA is the most prevalent protein in the analyzed pellicle of *B. amyloliquefaciens* l-17. In *B. subtilis* NCBI 3610, Romero et al. (2010) reported that TasA forms amyloid fibers which would provide structure to the extracellular matrix pellicle and, in conjunction with other EPSs components, hold the cells together. In the same strain, Steinberg et al. (2020) demonstrated that TasA acted as a signal stimulating a subset of biofilm cells to revert to a motile phenotype. Deletion of *tasA* has other effects, including the downregulation of genes related to sporulation and an increase in expression of EPSs matrix and antimicrobial secondary metabolite-related genes (Cámara-Almirón et al., 2020; Khambhati et al., 2021). A similar regulation may be expected in *B amyloliquefaciens* l-17 since flagellin and several sporulation-related proteins were identified in the pellicle matrix with the absence of gene products of the *eps*A–O operon, which oversees the production of EPSs matrix carbohydrates (Branda et al., 2001). However, the absence of the *eps*A–O operon products could also be linked to their loss during the analysis. Indeed, proteomics provides a global but non-exhaustive analysis of the proteins present in specific environments and time.

According to protein-protein interaction (STRING) parameters, TasA would interact with TapA protein (Fig. 3c). In this study, TapA (WP_015388007.1), the amyloid fiber anchoring/assembly protein TapA, was identified in the TB-fraction (Table 1). This protein forms a minor component of the TasA fibers and is required for their assembly in *B. subtilis* (Cairns et al., 2014). It has been reported that the *tapA* deletion strain is defective for pellicle formation and in the absence of *tapA,* TasA fibers cannot be formed and the level of TasA protein is reduced (Romero et al., 2011). The interaction network showed that TapA would interact with BslA protein (WP_003152178.1), one of the major proteins of the l-17 strain pellicle (Section 3.1). This last was described in *Bacillus* species as a major contributor to surface cohesiveness and water repellency for growing biofilms exposed to air (Kalamara et al., 2021; Morris et al., 2017). It also acts synergistically with TasA fibers and exopolysaccharide to generate structural complexity in the biofilm (Ostrowski et al., 2011). In addition, the interaction network emphasis the interaction between BslA and the extracellular triacylglycerol lipase (EstA, WP_003156672.1). However, the implication of this lipase in the biofilm formation is not described in the literature.

#### 3.2.4 Extracellular enzymes

Several extracellular enzymes were identified in this study (Table 1). Some exhibit interactions according to the prediction of STRING database (Fig. 3c). For example, the *aprE* gene product, S8 family serine peptidase (WP_003155195), was identified among the major proteins of LB-fraction (Fig. 2a). This protein exhibits 84 % sequence identity with its homologous subtilisin BPN' in *B. subtilis* 168. This serine protease is widely used in a variety in various industries including detergent, food processing and packaging, synthesis of inhibitory peptides, therapeutic, and waste management applications (Azrin et al., 2022).We also identified two other extracellular S8 family serine peptidase WP_015388265.1 and WP_014306056.1, homologous to Bpr and Vpr proteases of *B. subtilis* 168, respectively (Table 1 show the % of sequence identity). Vpr protease is one of the most abundant proteins in the TB-fraction, while Bpr protease was among the most prevalent proteins in the LB and TB fractions.

The abundance of proteases in the matrix and the predicted interaction network highlights the importance of these enzymes in the pellicle of *B. amyloliquefaciens* l-17. It's known that the extracellular proteases support growth of bacteria and play a crucial role in bacterial feeding: the degradation of cellular and environmental proteins as a source of amino acids and peptides (Harwood and Kikuchi, 2022; Rosazza et al., 2024). In *B. subtilis*, Rosazza et al. (2024) highlighted that the extracellular proteases promote colony biofilm formation and modulate its architecture, even when glutamic acid provides a freely available nitrogen source.

Other extracellular enzymes, such as chitosanase (WP_015387749.1), *csn* gene product which appears to be abundant in LB-fraction may be involved in the defense of the biofilm bacterial community against exogenous micro-organisms. In fact, the chitosanase isolated from *B. licheniformis* (Muslim et al., 2016) and *B. cereus* (Gao et al., 2008) exhibits an antibiofilm and antifungal activity, respectively.

The identification of the glycoside hydrolase family 68 protein (WP_014306184.1) homologous of the levansucrase encoded by *sacB* in *B. subtilis* 168 (Table 1) may suggest the probable presence of levan (homopolymer of fructose) in the EPSs polysaccharidic fraction in the pellicle matrix of *B. amyloliquefaciens* l-17. This enzyme is implicated in the levan biosynthesis (Benigar et al., 2014). Dogsa et al. (2013) showed that Levan, although not essential for biofilm formation, can be a structural and possibly stabilizing component of *B. subtilis* floating biofilms and may also serve as a nutritional reserve. The same hypothesis may be put forward in the pellicle of l-17 strain, but additional analyses are necessary to clarify the presence and the role of the Levan in *B. amyloliquefaciens* l-17 pellicle matrix.

#### 3.2.5 Spore related proteins

We identified about 10 proteins related to sporulation (Table 1) showing a predicted interaction on STRING map (Fig. 3c). Examples include CotE, CotR, CotF, CotJC, SpoVS, and SpoIVA. We have emphasized that the CotE and CotR proteins are among the 10 most abundant proteins in the TB-fraction (Fig. 2b) as of SopIVA protein in the LB-fraction (Fig. 2a).In addition, the following proteins: hypothetical protein (WP_021734090.1), FAD-binding oxidoreductase (WP_003156110.1), gamma-type small acid-soluble spore protein (WP_003155477.1), multicopper oxidase family protein (WP_003155789.1) and glycoside hydrolase family 18 protein (WP_032865795.1) showed strong homology with CotG, YvdP, SspE, CotA, and SleL of *B. subtilis* 168 (Table 1). The role of all these proteins in sporulation is well discussed in the literature (Hilbert and Piggot, 2004; McBride and Haldenwang, 2004; Mckenney et al., 2013; Riley et al., 2020). This suggests the presence of spores in the *B. amyloliquefaciens* l-17 pellicle. Overall, our results suggest cellular specialization and division of labor within the *B. amyloliquefaciens* l-17 pellicle, as reported in *B. subtilis* biofilm. Indeed, In the *B. subtilis* biofilm communities, different groups of cells fulfill distinct functions. Some bacteria produce EPSs while other members of a biofilm community can regain motility to explore the environment for new sources of nutrients. Furthermore, cells within the biofilm can differentiate into spores when the community gets older and nutrients are limiting (Gerwig and Stülke, 2014; Vlamakis et al., 2008). Little knowledge is available regarding the regulatory mechanisms that govern this sophisticated organization within the community.

### 3.3 Analysis of CFLP associated proteins

#### 3.3.1 Identification of CFLP associated proteins

**Fig. 4:**
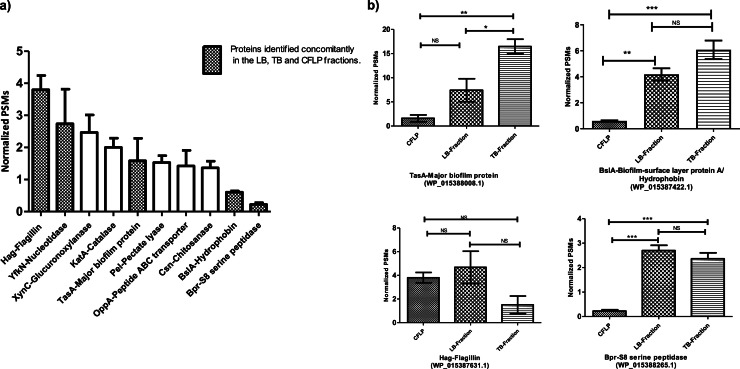
Label-free quantification based on spectral counting showing a) the abundance of the identified proteins in the cell free liquid phase (CFLP) of *B. amyloliquefaciens*l-17 and b) the differential abundance of TasA protein, BslA hydrophobin, flagellin and Bpr serine peptidase in the loosely bound (LB), tightly bound (TB) and CFLP fractions. Statistical significance for NS: no significative, ∗p < 0.05, ∗∗p < 0.01, and ∗∗∗p < 0.001. LB fraction contains proteins detached by mechanical disruption of the pellicle. TB fraction includes proteins chemically extracted from the pellicle.

During biofilm growth, the coexistence of planktonic and sessile cells can lead to dynamic exchanges between the two populations (El-Khoury et al., 2021). However, the interchanges between bacteria in these two states have been little explored. A molecular characterization of the liquid phase underlying the pellicle appears to be essential to decipher this coordination. In this study, we carried out for the first time a large-scale proteomic analysis of the CFLP of *B. amyloliquefaciens* l-17 (Fig. 1b). We identified 429 CFLP associated proteins (Table 2). Flagellin is the most abundant protein in CFLP (Fig. 4a). It is normal for planktonic *B. amyloliquefaciens* l-17 to produce this protein to build their flagella and ensure locomotion. Nevertheless, the over-presence of this protein in CFLP could suggest that a subpopulation of planktonic cells is propelled by flagella to tunnel deep within biofilm structure generating the biofilm irrigation and increasing nutrient flow in the matrix as it was demonstrated in *B. thuringiensis* 407 (Houry et al., 2012). Once again, we believe that confocal laser microscopy at regular intervals will be necessary to verify and explore this phenomenon in *B. amyloliquefaciens* strain l-17.

**Table 2:** Proteins identified in the cell free liquid phase (CFLP) of *B. amyloliquefaciens*l-17 and their cellular localization and biological functions. Proteins identified in both CFLP and pellicle (LB and TB fractions) are highlighted with a cross.

Accession N^a^	Protein Name^b^	Gene^c^	Molecular function^d^	Biological process^e^	Subcellular localization^f^	% Identity^g^ with *B. subtilis* 168 proteins	COG symbol^h^	CFLP^i^	LB-fraction^j^	TB-fraction^k^
***Amino acid transport and metabolism***										
WP_014305170.1	gamma-glutamyltransferase	*ggt*	glutathione hydrolase activity/ leukotriene C4 gamma-glutamyl transferase activity	proteolysis/ glutathione catabolic process	extracellular region	98	E	x	x	x
WP_003151173.1	F0F1 ATP synthase subunit beta	*pepA*	metalloaminopeptidase activity	proteolysis	cytoplasm	76	E	x		x
WP_015388449.1	peptide ABC transporter substrate-binding protein	*oppA*	peptide transmembrane transporter activity	protein transport/establishment of competence for transformation/sporulation	cell membrane	88	E	x	x	
WP_003154048.1	type I glutamate–ammonia ligase	*glnA*	Ligase (glu, ATP, nucluotid, Mg) metal binding	nitrogen catabolite repression of transcription/negative regulation of DNA-templated transcription/cellular response to nitrogen levels	unknown	97	E	x		x
WP_015388480.1	3-phosphoserine/phosphohydroxythreonine transaminase	*serC*	Aminotransferase/O-phospho-L‑serine:2-oxoglutarate aminotransferase activity/pyridoxal phosphate binding	amino-acid biosynthesis	cytoplasm	85	EH	x	x	
WP_003151992.1	leucyl aminopeptidase	*pepA*	metalloaminopeptidase activity	proteolysis	cytoplasm	76	E	x	x	
WP_003154169.1	aspartate-semialdehyde dehydrogenase	*comER*	pyrroline-5-carboxylate reductase activity	L-proline biosynthetic process	unknown	79	E	x	x	
WP_003154560.1	aminopeptidase	*ampS*	aminopeptidase activit	proteolysis	cytoplasm	87	E	x	x	
WP_003150810.1	peptidase T	*pepT*	tripeptide aminopeptidase activity	proteolysis	cytoplasm	78	E	x		
WP_015388345.1	peptide ABC transporter substrate-binding protein	*dppE*	peptide transmembrane transporter activity	protein transport/sporulation	cell membrane/periplasmic space	79	E	x		
WP_015388451.1	ornithine carbamoyltransferase	*argF*	ornithine carbamoyltransferase activity	arginine biosynthetic process via ornithine	cytoplasm	82	E	x		
WP_015388348.1	M55 family metallopeptidase	*dppA*	aminopeptidase activity	proteolysis/Sporulation	unknown	83	E	x		
WP_003153463.1	chorismate mutase	*aroH*	chorismate mutase activity	aromatic amino acid biosynthesis	cytoplasm	84	E	x		
WP_003156637.1	glycine/betaine ABC transporter	*opuAC*	transmembrane transporter activity	amino acid transport	cell membrane/membrane raft	85	E	x		
WP_015387510.1	5-methyltetrahydropteroyltriglutamate–homocysteine S-methyltransferase	*yxjG*	5-methyltetrahydropteroyltriglutamate-homocysteine S-methyltransferase activity	methionine biosynthetic process	unknown	86	E	x		
WP_014305305.1	tryptophan synthase subunit beta	*trpB*	tryptophan synthase activity	tryptophan biosynthetic process	cytoplasm	86	E	x		
WP_015388136.1	glutamate synthase large subunit	*gltA*	glutamate synthase activity	amino-acid biosynthesis	unknown	88	E	x		
WP_007611083.1	aminotransferase A	*dapX*	transaminase activity	lysine biosynthetic process via diaminopimelate	cytoplasm	88	E	x		
WP_015388703.1	4-aminobutyrate–2-oxoglutarate transaminase	*gabT*	4-aminobutyrate transaminase activit	gamma-aminobutyric acid catabolic process	cytoplasm	88	E	x		
WP_003153679.1	lysine 2,3-aminomutase	*kamA*	lysine 2,3-aminomutase activity	L-lysine catabolic process to acetate	unknown	89	E	x		
WP_015387777.1	alanine dehydrogenase	*ald*	alanine dehydrogenase activity	L-alanine catabolic process/sporulation	cytoplasm//plasma membrane	89	E	x		
WP_003156640.1	glycine/proline betaine ABC transporter ATP-binding protein OpuAA	*opuAA*	ABC-type quaternary ammonium compound transporting activity	amino acid transport	cell membrane	90	E	x		
WP_007408146.1	3-isopropylmalate dehydratase small subunit	*leuD*	3-isopropylmalate dehydratase activity	amino acid biosynthetic process	cytoplasm	92	E	x		
WP_003155800.1	2,3-butanediol dehydrogenase	*bdhA*	(R,R)-butanediol dehydrogenase activity	butanediol biosynthetic process	extracellular region/cytoplasm	93	E	x		
WP_015387765.1	threonine synthase	*thrC*	threonine synthase activity	amino-acid biosynthesis	cytoplasm	94	E	x		
WP_003151052.1	spermidine synthase	*speE*	spermidine synthase activity	spermidine biosynthetic process	cytoplasm	95	E	x		
WP_003151153.1	serine hydroxymethyltransferase	*glyA*	glycine hydroxymethyltransferase activity	amino-acid biosynthesis	cytoplasm	95	E	x		
WP_003152598.1	acetolactate synthase small subunit	*ilvH*	acetolactate synthase regulator activity	amino-acid biosynthesis	cytoplasm	98	E	x		
WP_015387606.1	acetolactate synthase AlsS	*alsS*	acetolactate synthase activity/flavin adenine dinucleotide binding		cytoplasm	84	EH	x		
WP_014304782.1	ABC transporter ATP-binding protein	*appD*	ATP hydrolysis activity	protein transport/establishment of competence for transformation/sporulation	plasma membrane	83	EP	x		
WP_003151199.1	urease subunit beta	*ureB*	urease activity	urea catabolic process	cytoplasm	76	E	x		
WP_003152866.1	late competence protein ComER	*comER*	pyrroline-5-carboxylate reductase activity	L-proline biosynthetic process	unknown	79	E	x		
WP_015387836.1	dipeptidase PepV		acetylornithine deacetylase activity	arginine biosynthetic process	unknown	80	E	x		
WP_003154692.1	cupin domain-containing protein	*mtnD*	acireductone dioxygenase [iron(II)-requiring] activity	methionine metabolic process	unknown	83	E	x		
WP_003152778.1	bifunctional cystathionine gamma-lyase/homocysteine desulfhydrase	*mccB*	cystathionine gamma-synthase activity	cysteine biosynthetic process via cystathionine/homocysteine catabolic process	cytoplasm	84	E	x		
WP_015388331.1	5-methyltetrahydropteroyltriglutamate–homocysteine S-methyltransferase	*metE*	5-methyltetrahydropteroyltriglutamate-homocysteine S-methyltransferase activity	amino-acid biosynthesis/Methylation	extracellular region/cytoplasm	89	E	x		
WP_014306158.1	arginase	*rocF*	arginase activity/manganese ion binding	arginine metabolic process/urea cycle	cytoplasm	91	E	x		
WP_003151938.1	homoserine dehydrogenase	*hom*	homoserine dehydrogenase activity	amino-acid biosynthesis	cytoplasm/ extracellular region	93	E	x		
WP_003153209.1	branched-chain amino acid dehydrogenase	*yqiT*	glutamate dehydrogenase [NAD(P)+] activity/leucine dehydrogenase activity	leucine catabolic process	unknown	94	E	x		
WP_015387842.1	bifunctional 3-deoxy-7-phosphoheptulonate synthase/chorismate mutase	*aroA*	3-deoxy-7-phosphoheptulonate synthase activity	aromatic amino acid family biosynthetic process	unknown	95	E	x		
WP_015388257.1	carbamoyl-phosphate synthase (glutamine-hydrolyzing) large subunit	*pyrAB*	aspartate carbamoyltransferase activity/ ATP binding	amino-acid biosynthesis/Pyrimidine biosynthesis	cytoplasm	92	EF	x		
WP_015388490.1	D-amino-acid transaminase	*dat*	D-alanine:2-oxoglutarate aminotransferase activity	D-amino acid biosynthetic process	cytoplasm	74	EH	x		
WP_003152600.1	ketol-acid reductoisomerase	*ilvC*	ketol-acid reductoisomerase activity/magnesium ion binding/NADP binding	amino-acid biosynthesis	cytoplasm	96	EH	x		
***Carbohydrate transport and metabolism***										
WP_015387727.1	oxalate decarboxylase family bicupin	*oxdC*	oxalate decarboxylase activity	oxalate metabolic proces	periplasmic space/cytoplasm	87	G	x	x	x
WP_021734196.1	M42 family metallopeptidase	*yhfE*	aminopeptidase activity	proteolysis	unknown	83	GE	x	x	
WP_003150769.1	glycoside hydrolase family 16 protein	*ydhD*	glycosidase/Hydrolase/chitin binding	carbohydrate metabolism/polysaccharide degradation/sporulation	extracellular region/spore wall	72	G	x	x	
WP_015388558.1	pectate lyase	*pel*	pectate lyase activity/metal ion binding	pectin catabolic process	extracellular region	76	G	x	x	
WP_015388729.1	starch-binding protein	*amyE*	alpha-amylase activity/ glycosidase/hydrolase	Carbohydrate metabolism	extracellular region	86	G	x	x	
WP_015388167.1	carbohydrate-binding protein	*xynD*	glycosidase/hydrolase	carbohydrate metabolism/polysaccharide degradation/xylan degradation	extracellular region	90	G	x	x	
WP_003151102.1	fructose-bisphosphate aldolase	*fbaA*	fructose-bisphosphate aldolase activity/tagatose-bisphosphate aldolase activity	lyase/glycolysis/sporulation	cytoplasm	99	G	x	x	
WP_015388172.1	cellulase family glycosylhydrolase	*eglS*	beta-glucosidase activity/cellulase activity	cellulose catabolic process	extracellular region/Cell surface	94	G	x	x	
WP_003151103.1	fructose-6-phosphate aldolase	*tal*	aldehyde-lyase activity	carbohydrate metabolic process	cytoplasm	90	G	x	x	
WP_003151611.1	type I glyceraldehyde-3-phosphate dehydrogenase	*gapA*	oxidoreductase/NADP binding	glycolysis	unknown	97	G	x		x
WP_015387871.1	glycoside hydrolase family 43 protein	*abnA*	arabinan endo-1,5-alpha-l-arabinosidase activity	arabinan catabolic process	extracellular region	84	G	x		
WP_021734251.1	carbohydrate kinase		kinase activity	phosphorylation	unknown	88	G	x		
WP_015388180.1	transketolase	*tkt*	transketolase activity	pentose-phosphate shunt	cytoplasm	90	G	x		
WP_007408647.1	glucose-6-phosphate isomerase	*pgi*	glucose-6-phosphate isomerase activity	glycolytic process	cytoplasm	94	G	x		
WP_003156759.1	phosphoglucosamine mutase	*glmM*	phosphoglucosamine mutase activity	peptidoglycan biosynthetic process	cytoplasm	95	G	x		
WP_007408096.1	M42 family metallopeptidase	*ysdC*	aminopeptidase activity	proteolysis	unknown	90	GE	x		
WP_003152432.1	metal-dependent hydrolase	*ytkL*	hydrolase activity	unknown	unknown	92	GE	x		
WP_015388184.1	peptidase G2	*yobO*		unknown	unknown	81	G	x		
WP_003154656.1	glucose-specific PTS transporter subunit IIBC	*ptsG*	D-glucosamine PTS permease activity/glucose transmembrane transporter activity/kinase activity	phosphoenolpyruvate-dependent sugar phosphotransferase system	plasma membrane	93	G	x		
WP_003151620.1	2,3-bisphosphoglycerate-independent phosphoglycerate mutase	*gpmI*	2,3-bisphosphoglycerate-independent phosphoglycerate mutase activity	sporulation/glycolysis	cytoplasm	96	G	x		
WP_003151616.1	phosphoglycerate kinase	*pgk*	phosphoglycerate kinase activity	glycolysis/phosphorylation	cytoplasm	98	G	x		
WP_003151618.1	triose-phosphate isomerase	*tpiA*	triose-phosphate isomerase activity	gluconeogenesis/glycerol catabolic process	cytoplasm	98	G	x		
WP_003153241.1	NADP-dependent phosphogluconate dehydrogenase	*gndA*	phosphogluconate 2-dehydrogenase activity/NADP binding	pentose-phosphate shunt, oxidative branch	unknown	99	G	x		
WP_007409956.1	phosphopyruvate hydratase	*eno*	phosphopyruvate hydratase complex	sporulation/Glycolysis	extracellular region/cytoplasm	99	G	x		
WP_003152370.1	M42 family metallopeptidase	*ytoP*	metallopeptidase activity	proteolysis	unknown	88	GE	x		
***Cell cycle control, cell division, chromosome partitioning, Inorganic ion transport and metabolism, Cell wall/membrane/envelope biogenesis***										
WP_015387430.1	spore coat protein	*cotF*	Unknown	sporulation	spore coat	71	D	x	x	x
WP_015388201.1	N-acetylmuramoyl-l-alanine amidase	*cwlC*	N-acetylmuramoyl-l-alanine amidase activity/peptidoglycan binding/hydrolase	peptidoglycan catabolic process/sporulation	extracellular region/spore wall	77	D	x	x	x
WP_003155789.1	multicopper oxidase family protein	*cotA*	oxidoreductase	sporulation	periplasmic space	77	D	x		x
WP_003155477.1	gamma-type small acid-soluble spore protein	*sspE*	unknown	sporulation	unknown	84	D	x		x
WP_003153438.1	stage IV sporulation protein A	*spoIVA*	ATP hydrolysis activity/	sporulation	spore wall/endospore cortex	96	D	x		x
WP_003155170.1	hypothetical protein	*spoIVA*	ATP hydrolysis activity/	sporulation	spore wall/endospore cortex	96	D	x	x	
WP_015388222.1	DUF4115 domain-containing protein	*ymfM*	DNA binding	unknown	plasma membrane	72	D	x		
WP_015387885.1	GerMN domain-containing protein	*gerM*	Unknown	sporulation	Unknown	82	D	x		
WP_014304355.1	penicillin-binding transpeptidase domain-containing protein	*pbpC*	peptidoglycan L,D-transpeptidase activity/penicillin binding	cell wall organization/regulation of cell shape	plasma membrane	76	DM	x		
WP_003152647.1	cell shape-determining protein MreB	*mreB*	ATP binding	cell morphogenesis	cytoplasm	90	D	x		
WP_003154424.1	cell division protein FtsZ	*ftsZ*	GTPase activity	cell division	cytoplasm	95	D	x		
***Cell motility***										
WP_003154244.1	flagellar basal body rod protein FlgG	*flgG*	bacterial-type flagellum assembly	bacterial-type flagellum-dependent swarming motility	bacterial flagellum	72	N	x		
***Cell wall/membrane/envelope biogenesis***										
WP_015388671.1	hypothetical protein	*yxaL*	unknown	unknown	extracellular region	74	M	x		x
WP_003155677.1	LTA synthase family protein	*ltaS1*	metal ion binding	cell wall organization	extracellular region/plasma membrane	85	M	x	x	
WP_003154166.1	4‑hydroxy-tetrahydrodipicolinate synthase	*dapA*	4‑hydroxy-tetrahydrodipicolinate synthase activity	mino-acid biosynthesis	cytoplasm	94	ME	x		x
WP_003150773.1	DUF3298 and DUF4163 domain-containing protein	*yxiT*	unknown	unknown	unknown	84	M	x	x	
WP_003154742.1	glycoside hydrolase family 18 protein	*ydhD*	chitin binding chitin activity	chitin catabolic process/Carbohydrate metabolism/sporulation	extracellular region	72	M	x	x	
WP_032865795.1	glycoside hydrolase family 18 protein	*sleL*	glycosidase Hydrolase	sporulation	spore coat	78	M	x	x	
WP_015388752.1	DUF1906 domain-containing protein	*ybfG*	unknown	unknown	cell membrane	84	M	x	x	
WP_015387749.1	chitosanase	*csn*	glycosidase/Hydrolase	carbohydrate metabolic process	extracellular region	88	M	x	x	
WP_015388168.1	glucuronoxylanase xynC	*xynC*	glycosidase/Hydrolase	carbohydrate metabolism/xylan degradation/polysaccharide degradation/ sphingolipid metabolic process	extracellular region	98	M	x	x	
WP_088005523.1	PQQ-binding-like beta-propeller repeat protein	*yxaL*	Increases the processivity of the PcrA helicase, but does not bind to DNA.		extracellular region	76	M	x	x	
WP_015388018.1	alanine racemase	*alr2*	catalyzes the interconversion of l-alanine and d-alanine. pyridoxal phosphate binding	D-alanine biosynthetic process/ peptidoglycan biosynthetic process	cytoplasm	84	M	x	x	
WP_003151206.1	endo-1,4-beta-xylanase XynA	*xynA*	glycosidase/Hydrolase	carbohydrate metabolism/Polysaccharide degradation/Xylan degradation	unknown	95	MG	x		x
WP_015388080.1	PBP1A family penicillin-binding protein	*ponA*	penicillin binding/peptidoglycan glycosyltransferase activity	peptidoglycan biosynthetic process/response to antibiotic	unknown	74	M	x		
WP_003151892.1	SIS domain-containing protein	*frlB*	glutamine-fructose-6-phosphate transaminase (isomerizing) activity	carbohydrate metabolism	unknown	86	M	x		
WP_015388267.1	UDP-N-acetylmuramate dehydrogenase	*murB*	UDP-N-acetylmuramate dehydrogenase activity/flavin adenine dinucleotide binding	cell division/cell cycle/cell shape	cytoplasm	87	M	x		
WP_003154428.1	undecaprenyldiphospho-muramoylpentapeptide beta-N-acetylglucosaminyltransferase	*murG*	glycosyltransferase activity	cell cycle/cell division/cell shape/peptidoglycan synthesis	cytoplasm	90	M	x		
WP_003152281.1	ABC transporter ATP-binding protein	*ytrE*	transmembrane transporter activity	transport	cell membrane/ peripheral membrane protein	92	M	x		
WP_003154436.1	UDP-N-acetylmuramoyl-l-alanyl-d-glutamate–2,6-diaminopimelate ligase	*murE*	UDP-N-acetylmuramoylalanyl-d-glutamate-2,6-diaminopimelate ligase activity	cell cycle/cell division/cell shape/peptidoglycan synthesis	cytoplasm		M	x		
WP_014304858.1	N-acetylmuramoyl-l-alanine amidase	*xlyA*	N-acetylmuramoyl-l-alanine amidase activity	peptidoglycan catabolic process/sporulation	extracellular region	78	M	x		
WP_003150715.1	D-alanyl-d-alanine carboxypeptidase	*dacA*	serine-type d-Ala-d-Ala carboxypeptidase activity	cell wall organization/peptidoglycan biosynthetic process	extracellular region/plasma membrane	80	M	x		
WP_003156191.1	outer membrane lipoprotein carrier protein LolA	*ydcC*	unknown	sporulation	plasma membrane	88	M	x		
WP_015387544.1	dTDP-4-dehydrorhamnose 3,5-epimerase family protein	*spsL*	dTDP-4-dehydrorhamnose 3,5-epimerase activity	extracellular polysaccharide biosynthetic process/dTDP-rhamnose biosynthetic process	cytoplasm	90	M	x		
WP_003154355.1	extracellular matrix/biofilm regulator RemA	*remA*	DNA-binding	Transcription	unknown	100	ME	x		
***Coenzyme transport and metabolism***										
WP_015387611.1	Cof-type HAD-IIB family hydrolase	*YwtE*	magnesium ion binding/phosphatase activity	riboflavin biosynthetic process	cytoplasm	77	H	x		
WP_015387992.1	Nif3-like dinuclear metal center hexameric protein	*yqfO*	metal ion binding	unknown	cytoplasm	82	H	x		
WP_003155224.1	heme-degrading oxygenase HmoB	*hmoB*	heme oxygenase (decyclizing) activity	metal ion binding	cytoplasm	84	H	x		
WP_015388476.1	ferrochelatase	*cpfC*	ferrochelatase activity	heme biosynthetic process	cytoplasm	84	H	x		
WP_003150671.1	dihydroneopterin aldolase	*folB*	dihydroneopterin aldolase activity	tetrahydrofolate biosynthetic process	cytoplasm	89	H	x		
WP_003153510.1	aspartate 1-decarboxylase	*panD*	aspartate 1-decarboxylase activity	pantothenate biosynthetic process	cytoplasm	92	H	x		
WP_015388486.1	Cof-type HAD-IIB family hydrolase	*yhaX*	magnesium ion binding/phosphatase activity	stress response	cytoplasm	87	H	x		
WP_015388517.1	phosphomethylpyrimidine synthase ThiC	*thiC*	4 iron, 4 sulfur cluster binding	thiamine biosynthetic process	cytoplasm	91	H	x		
WP_003153372.1	6,7-dimethyl-8-ribityllumazine synthase	*ribH*	6,7-dimethyl-8-ribityllumazine synthase activity	riboflavin biosynthetic process	cytoplasm	93	H	x		
WP_003153401.1	phosphoglycerate dehydrogenase	*serA*	formate dehydrogenase (NAD+) activity/phosphoglycerate dehydrogenase activity	L‑serine biosynthetic process	cytoplasm	94	H	x		
WP_003152427.1	molybdenum cofactor biosynthesis protein MoaB	*moaB*	unknown	molybdenum cofactor biosynthesis	cytoplasm	82	HP	x		
WP_003151011.1	heme-dependent peroxidase	*chdC*	oxidoreductase/metal ion binding/peroxidase activity	heme biosynthetic process	unknown	94	HP	x		
WP_003152221.1	1,4-dihydroxy-2-naphthoyl-CoA synthase	*menB*	1,4-dihydroxy-2-naphthoyl-CoA synthase activity	menaquinone biosynthetic process	cytoplasm	96	HP	x		
WP_003152253.1	methionine adenosyltransferase	*metK*	methionine adenosyltransferase activity/ magnesium ion binding	S-adenosylmethionine biosynthetic process	cytoplasm	98	HP	x		
***Defense mechanisms***										
WP_004393188.1	peroxiredoxin	*ahpC*	antioxydant/Oxidoreductase/Peroxidase/Stress response	hydrogen peroxide catabolic process/response to oxidative stress	cytoplasm	98	V	x	x	x
WP_015387507.1	catalase	*katE*	catalase activity/heme binding	hydrogen peroxide catabolic process/response to oxidative stress	cytoplasm	84	V	x	x	
WP_139,886,654.1	class A beta-lactamase	*penP*	hydrolase	antibiotic resistance	unknown	91	V	x	x	
WP_007614902.1	alkyl hydroperoxide reductase subunit F	*ahpF*	oxidoreductase/Translocase	electron transport	cell membrane	95	V	x	x	
WP_152,514,787.1	multifunctional 2′,3′-cyclic-nucleotide 2′-phosphodiesterase/3′-nucleotidase/5′-nucleotidase	*yfkN*	metal ion binding/nucleotide binding	nucleotide catabolic process	extracellular region/ periplasmic space	74	VF	x		x
WP_014305062.1	dUTP diphosphatase	*yncF*	dUTP diphosphatase activity/magnesium ion binding	nucleotide metabolism	unknown	87	VF	x		
WP_015388087.1	glutathione peroxidase	*bsaA*	phospholipid-hydroperoxide glutathione peroxidase activity	cellular response to oxidative stress	cytoplasm	84	VI	x		
WP_003151815.1	DNA starvation/stationary phase protection protein	*mrgA*	DNA-binding/oxidoreductase activity, acting on metal ions	stress response	unknown	75	VP	x		
WP_003152242.1	DNA starvation/stationary phase protection protein	*dps*	oxidoreductase activity, acting on metal ions	stress response	unknown	91	VP	x		
WP_003154758.1	organic hydroperoxide resistance protein	*ohrB*	unknown	stress response	unknown	81	V	x		
WP_003152408.1	GAF domain-containing protein	*ytsP*	unknown	unknown	cytoplasm	87	VT	x		
***Energy production and conversion***										
WP_003152455.1	malate dehydrogenase	*mdh*	oxidoreductase	tricarboxylic acid cycle	cytoplasm	96	C	x	x	
WP_003151167.1	F0F1 ATP synthase subunit C	*atpE*	proton-transporting ATP synthase activity, rotational mechanism/ lipid binding	ATP synthesis/Hydrogen /ion transporton transport	plasma membrane	100	C	x	x	
WP_015388516.1	FAD-binding oxidoreductase	*ygaK*	oxidoreductase activity/ FAD binding	unknown	unknown	85	C	x		x
WP_003156110.1	FAD-binding oxidoreductase	*yvdP*	oxidoreductase	sporulation	spore coat	98	C	x		x
WP_015388129.1	molybdopterin oxidoreductase family protein	*yoaE*	iron-sulfur cluster binding	unknown	unknown	80	C	x		
WP_003154530.1	pyruvate dehydrogenase (acetyl-transferring) E1 component subunit alpha	*pdhA*	pyruvate dehydrogenase (acetyl-transferring) activity	acetyl-CoA biosynthetic process from pyruvate	unknown	86	C	x		
WP_003156323.1	antibiotic biosynthesis monooxygenase	*ycnE*	monooxygenase activity	aromatic compound catabolic process/response to toxic substance	cytoplasm	88	C	x		
WP_003153791.1	FMN-dependent NADH-azoreductase	*azoR1*	oxidoreductase activity, acting on NAD(P)H, NAD(P) as acceptor	aromatic hydrocarbons catabolism/response to toxic substance	unknown	90	C	x		
WP_003153484.1	ubiquinol-cytochrome c reductase iron-sulfur subunit	*qcrA*	2 iron, 2 sulfur cluster binding	oxidoreductase activity	plasma membrane	91	C	x		
WP_003154490.1	pyruvate carboxylase	*pyc*	pyruvate carboxylase activity	pyruvate metabolic process	cytoplasm	91	C	x		
WP_014305704.1	iron-containing alcohol dehydrogenase	*yugJ*	alcohol dehydrogenase (NADP+) activity	methylglyoxal reductase (NADPH-dependent, acetol producing)	cytoplasm	92	C	x		
WP_015388115.1	2-oxoglutarate dehydrogenase E1 component	*odhA*	oxoglutarate dehydrogenase complex	tricarboxylic acid cycle	cytoplasm	92	C	x		
WP_015388116.1	2-oxoglutarate dehydrogenase complex dihydrolipoyllysine-residue succinyltransferase	*odhB*	dihydrolipoyllysine-residue succinyltransferase activity	tricarboxylic acid cycle	cytoplasm	92	C	x		
WP_003151719.1	FMN-dependent NADH-azoreductase AzoRB	*azoR2*	electron transfer activity	response to toxic substance/aromatic compound catabolic process	unknown	94	C	x		
WP_003155386.1	NAD(P)H-dependent oxidoreductase	*yhcB*	unknown	NAD(P)H dehydrogenase (quinone) activity	cell membrane	94	C	x		
WP_003152575.1	succinate dehydrogenase iron-sulfur subunit	*sdhB*	electron transfer activity	tricarboxylic acid cycle/Electron transport	plasma membrane	94	C	x		
WP_003154907.1	NAD(P)/FAD-dependent oxidoreductase	*yjlD*	NAD(P)H dehydrogenase (quinone) activity	aerobic electron transport chain	unknown	95	C	x		
WP_003152573.1	succinate dehydrogenase flavoprotein subunit	*sdhA*	electron transfer activity	tricarboxylic acid cycle/Electron transport	plasma membrane	96	C	x		
WP_003151170.1	F0F1 ATP synthase subunit alpha	*atpA*	ATP binding	proton motive force-driven ATP synthesis	plasma membrane	98	C	x		
WP_014304945.1	dihydrolipoyl dehydrogenase	*pdhD*	dihydrolipoyl dehydrogenase activity	unknown	cytoplasm	98	C	x		
WP_015387882.1	electron transfer flavoprotein subunit alpha/FixB family protein	*etfA*	specific electron acceptor for other dehydrogenases		unknown	81	C	x		
WP_015388029.1	dihydrolipoyl dehydrogenase	*bfmBC*	dihydrolipoyl dehydrogenase activity/flavin adenine dinucleotide binding		cytoplasm	83	C	x		
WP_015388104.1	nitroreductase family protein	*yodC*	oxidoreductase activity	aromatic compound catabolic process/response to toxic substance	cytoplasm	84	c	x		
WP_003151012.1	phosphate acetyltransferase	*pta*	phosphate acetyltransferase activity	acetyl-CoA biosynthetic process	cytoplasm	93	C	x		
WP_014305071.1	aconitate hydratase AcnA	*citB*	2-methylisocitrate dehydratase activity/4 iron, 4 sulfur cluster binding/aconitate hydratase activity/iron-responsive element binding/mRNA 3′-UTR binding	citrate metabolic process/propionate metabolic process, methylcitrate cycle/regulation of sporulation/tricarboxylic acid cycle	cytoplasm	93	C	x		
WP_015387863.1	NADP-dependent isocitrate dehydrogenase	*icd*	isocitrate dehydrogenase (NADP+) activity/ magnesium ion binding	glyoxylate bypass/Tricarboxylic acid cycle	unknown	94	C	x		
WP_014305566.1	citrate synthase	*citZ*	citrate (Si)-synthase activity	carbohydrate metabolic process/ tricarboxylic acid cycle	cytoplasm	94	C	x		
WP_003154526.1	pyruvate dehydrogenase complex dihydrolipoyllysine-residue acetyltransferase	*pdhC*	dihydrolipoyllysine-residue succinyltransferase activity	tricarboxylic acid cycle	cytoplasm	96	C	x		
WP_003154281.1	succinate–CoA ligase subunit alpha	*sucD*	succinate-CoA ligase (GDP-forming) activity	tricarboxylic acid cycle	cytoplasm	97	C	x		
WP_003154283.1	ADP-forming succinate–CoA ligase subunit beta	*sucC*	succinate-CoA ligase (GDP-forming) activity	tricarboxylic acid cycle	cytoplasm	98	C	x		
***Inorganic ion transportand metabolism***										
WP_012118382.1	methionine ABC transporter substrate-binding lipoprotein MetQ	*metQ*	unknown	amino-acid transport	cell membrane	87	P	x	x	x
WP_003155420.1	catalase KatA	*katA*	oxidoreductase/Peroxidase	hydrogen peroxide catabolic process	cytoplasm	94	P	x	x	x
WP_014305921.1	right-handed parallel beta-helix repeat-containing protein	*ywoF*	unknown	unknown	extracellular region	74	P	x	x	
WP_003156656.1	metal ABC transporter substrate-binding protein	*znuA*	metal ion binding	zinc ion transport, cell adhesion	plasma membrane	83	P	x		x
WP_003156778.1	ABC transporter substrate-binding protein	*feuA*	part of the ABC transporter complex FeuABC/YusV involved in import of the catecholate siderophores bacillibactin and enterobactin.	iron ion transport	cytoplasm/ cell membrane	83	P	x	x	
WP_003155700.1	spore coat protein CotJC	*cotJC*	spore coat composition	sporulation	unknown	96	P	x		x
WP_004393259.1	iron-hydroxamate ABC transporter substrate-binding protein	*yxeB*	unknown	iron ion transport	plasma membrane/periplasmic space	72	P	x		
WP_003153772.1	superoxide dismutase	*sodF*	superoxide dismutase activity	unknown	unknown	80	P	x		
WP_007408225.1	formate/nitrite transporter family protein	*yrhG*	formate transmembrane transporter activity	formate transport/nitrite transport	plasma membrane	84	P	x		
WP_015388253.1	sulfate adenylyltransferase	*sat*	sulfate adenylyltransferase (ATP) activity	sulfate assimilation	unknown	87	P	x		
WP_015387719.1	assimilatory sulfite reductase (NADPH) hemoprotein subunit	*cysI*	sulfite reductase complex (NADPH)	cysteine biosynthetic process/amino-acid biosynthesis	cytoplasm	90	P	x		
WP_003153025.1	superoxide dismutase SodA	*sodA*	superoxide dismutase activity/metal ion binding	removal of superoxide radicals	cytoplasm	92	P	x		
***Intracellular trafficking, secretion, and vesicular transport***										
WP_003151414.1	preprotein translocase subunit SecA	*secA*	protein-exporting ATPase activity	intracellular protein transmembrane transport	cell membrane/ cell envelope Sec protein transport complex	94	U	x		
***Lipid transport and metabolism***										
WP_003156672.1	triacylglycerol lipase	*estA*	triglyceride lipase activity	lipid catabolic process	extracellular region	76	I	x		x
WP_003156315.1	glucose 1-dehydrogenase	*gdh*	oxidoreductase/glucose 1-dehydrogenase [NAD(P)] activity	sporulation	unknown	90	I	x	x	
WP_003155182.1	glucose 1-dehydrogenase	*yhxC*	oxidoreductase	unknown	unknown	92	I	x		x
WP_003154310.1	acyl carrier protein	*acpA*	carrier of the growing fatty acid chain in fatty acid biosynthesis.	fatty acid metabolism	cytoplasm	100	I	x		x
WP_003152442.1	acetyl-CoA carboxylase carboxyl transferase subunit alpha	*accA*	carboxyl- or carbamoyltransferase activity	fatty acid biosynthetic process/response to antibiotic	cytoplasm	88	I	x		
WP_003153776.1	aldehyde dehydrogenase family protein	*dhaS*	phenylacetaldehyde dehydrogenase activity	cellular aldehyde metabolic process	unknown	90	I	x		
WP_003152391.1	acetate–CoA ligase	*acsA*	acetate-CoA ligase activity	acetyl-CoA biosynthetic process	cytoplasm/cell membrane	90	I	x		
WP_003153973.1	YbgC/FadM family acyl-CoA thioesterase	*yneP*	fatty acyl-CoA hydrolase activity	unknown	unknown	92	I	x		
WP_003156602.1	L-glutamate gamma-semialdehyde dehydrogenase	*putC*	1-pyrroline-5-carboxylate dehydrogenase activity	proline catabolic process to glutamate	plasma membrane	97	I	x		
WP_003155056.1	beta-ketoacyl-ACP synthase II	*fabF*	3-oxoacyl-[acyl-carrier-protein] synthase activity	fatty acid biosynthetic process	cytoplasm	92	IQ	x		
WP_014305231.1	3-phytase	*phy*	3-phytase activity	unknown	extracellular region	72	I	x		
WP_015388475.1	beta-ketoacyl-ACP synthase III FabHB	*fabHB*	3-oxoacyl-[acyl-carrier-protein] synthase activity	fatty acid biosynthetic process/secondary metabolite biosynthetic process	cytoplasm	80	I	x		
WP_003155001.1	enoyl-ACP reductase FabI	*fabI*	enoyl-[acyl-carrier-protein] reductase (NADH) activity	cellular response to cold/fatty acid elongation	unknown	95	I	x		
WP_003156407.1	2-C-methyl-d-erythritol 2,4-cyclodiphosphate synthase	*ispF*	2-C-methyl-d-erythritol 2,4-cyclodiphosphate synthase activity	isopentenyl diphosphate biosynthetic process, methylerythritol 4-phosphate pathway	unknown	95	I			
***Mobilome: prophages, transposons***							x			
WP_007407274.1	phage major capsid protein	*xkdG*	Possibly a prophage capsid protein.	unknown	extracellular region	91	X	x		
***Nucleotide transport and metabolism***										
WP_003150717.1	IMP dehydrogenase	*guaB*	oxydoreductase	GMP biosynthesis/Purine biosynthesis	cytoplasm	96	F	x	x	
WP_015387718.1	assimilatory sulfite reductase (NADPH) flavoprotein subunit	*cysJ*	flavin adenine dinucleotide binding/FMN binding	cysteine biosynthetic process/amino-acid biosynthesis	cytoplasm	78	FP	x	x	
WP_003155232.1	HIT family protein	*hit*	catalytic activity	nucleotide metabolic process	unknown	85	F	x		
WP_003153455.1	nucleoside-diphosphate kinase	*ndk*	nucleoside diphosphate kinase activity	nucleotide metabolism	cytoplasm	90	F	x		
WP_003153486.1	menaquinol-cytochrome c reductase cytochrome b/c subunit	*ndk*	nucleoside diphosphate kinase activity	nucleotide metabolism	cytoplasm	90	F	x		
WP_015388096.1	purine-nucleoside phosphorylase	*deoD*	purine-nucleoside phosphorylase activity	purine nucleoside catabolic process	cytoplasm	90	F	x		
WP_015387764.1	TIGR01457 family HAD-type hydrolase	*yutF*	alkaline phosphatase activity	unknown	cytoplasm	91	F	x		
WP_003154392.1	bifunctional pyrimidine operon transcriptional regulator/uracil phosphoribosyltransferase	*pyrR*	uracil phosphoribosyltransferase activity	DNA-templated transcription termination	plasma membrane	94	F	x		
WP_004393102.1	adenylosuccinate synthase	*purA*	adenylosuccinate synthase activity	IMP metabolic process	cytoplasm	94	F	x		
WP_003155784.1	glutamine-hydrolyzing GMP synthase	*guaA*	GMP synthase activity	GMP biosynthetic process	cytoplasm	97	F	x		
WP_004264656.1	ribose-phosphate diphosphokinase	*prs*	kinase activity	nucleotide biosynthesis	cytoplasm	95	F	x		
WP_007410407.1	adenylate kinase	*adk*	adenylate kinase activity	nucleoside diphosphate metabolic process	cytoplasm	95	F	x		
***Posttranslation modification, protein turnover, chaperones***										
WP_004264608.1	ATP-dependent zinc metalloprotease FtsH	*ftsH*	ATP-dependent peptidase activity	protein catabolic process/cell cycle/sporulation	plasma membrane	96	O	x	x	x
WP_015388265.1	S8 family serine peptidase	*bpr*	serine-type endopeptidase activity	proteolysis	extracellular region	70	O	x	x	x
WP_003155497.1	serine protease	*mpr*	serine-type endopeptidase activity	proteolysis	extracellular region	74	O	x	x	
WP_015388283.1	M4 family metallopeptidase	*nprE*	metalloendopeptidase activity	proteolysis	extracellular region	83	O	x	x	
WP_003154079.1	S8 family peptidase	*aprX*	serine-type endopeptidase activity	proteolysis	extracellular region/cytoplasm	83	O	x	x	
WP_014304883.1	S8 family peptidase	*isp*	serine-type endopeptidase activity	proteolysis	unknown	93	O	x	x	
WP_014305002.1	HslU–HslV peptidase ATPase subunit	*clpY*	ATP hydrolysis activity	proteolysis	unknown	93	O	x	x	
WP_003155249.1	peptidylprolyl isomerase	*prsA*	protein folding and transport	peptidyl-prolyl cis-trans isomerase activity	plasma membrane	85	O	x		x
WP_003155195.1	S8 family serine peptidase	*aprE*	serine protease	fibrinolysis/proteolysis/sporulation	extracellular region	85	O	x	x	
WP_007410388.1	ATP-dependent protease ATP-binding subunit ClpC	*clpC*	ATP hydrolysis activity/Chaperone/Repressor	chaperone/establishment of competence for transformation	cytoplasm	98	O	x		x
WP_015387737.1	trypsin-like peptidase domain-containing protein	*htrB*	serine-type endopeptidase activity	proteolysis	plasma membrane	76	O	x		
WP_003153552.1	carboxypeptidase M32	*ypwA*	metallocarboxypeptidase activity	proteolysis	unknown	83	O	x		
WP_003153641.1	peptide-methionine (*S*)-S-oxide reductase MsrA	*msrA*	peptide-methionine (*S*)-S-oxide reductase activity	protein modification process	unknown	87	O	x		
WP_003156398.1	protein arginine kinase	*mcsB*	kinase activity	phosphorylation	extracellular region/cytoplasm	91	O	x		
WP_015387840.1	thioredoxin family protein	*ytpP*	unknown	unknown	unknown	92	O	x		
WP_007408050.1	thiol peroxidase	*tpx*	thioredoxin peroxidase activity	unknown	unknown	94	O	x		
WP_003151513.1	ATP-dependent Clp endopeptidase proteolytic subunit ClpP	*clpP*	ATP-dependent peptidase activity	protein quality control for misfolded or incompletely synthesized proteins/Stress response	cytoplasm	96	O	x		
WP_003152893.1	molecular chaperone DnaK	*dnaK*	ATP-dependent protein folding chaperone	protein refolding/stress response	cytoplasm	97	O	x		
WP_015388098.1	S41 family peptidase	*ctpA*	serine-type endopeptidase activity	proteolysis//signal transduction	periplasmic space	81	O	x		
WP_015387889.1	ATP-dependent protease ATP-binding subunit ClpX	*clpX*	ATP hydrolysis activity/ATP-dependent protein folding chaperone	protein catabolic process/ cell division	unknown	96	O	x		
WP_003152618.1	trigger factor	*tig*	protein folding chaperone/peptidyl-prolyl cis-trans isomerase activity	cell division/Cell cycle	cytoplasm	96	O	x		
WP_003155941.1	chaperonin GroEL	*groEL*	GroEL-GroES complex/ATP-dependent protein folding chaperone	protein refolding/response to heat	extracellular region/Cytoplasme	98	O	x		
WP_003152560.1	thioredoxin	*trxA*	protein-disulfide reductase activity	cell redox homeostasis	cytoplasm	99	O	x		
***Replication, recombination and repair***										
WP_003153447.1	non-specific DNA-binding protein Hbs	*hbs*	DNA binding	chromosome condensation	cytoplasm	100	L	x		x
WP_004392898.1	DNA gyrase subunit A	*gyrA*	DNA topoisomerase type II (double strand cut, ATP-hydrolyzing) complex	DNA topological change	cytoplasm	93	L	x		
WP_004392911.1	DNA polymerase III subunit beta	*dnaN*	3′−5′ exonuclease activity	DNA strand elongation involved in DNA replication	cytoplasm	94	L	x		
WP_003154145.1	recombinase RecA	*RecA*	ATP-dependent DNA damage sensor activity/damaged DNA binding	unknown	unknown	96	L	x		
WP_003151902.1	ribonuclease	*bsn*	nuclease activity	unknown	extracellular region	78	L	x		
WP_015388679.1	ATP-dependent RNA helicase CshA	*cshA*	ATP hydrolysis activity/RNA helicase activity	bibosome biogenesis/Stress response	cytoplasm/ plasma membrane	97	L	x		
WP_004392979.1	single-stranded DNA-binding protein SsbA	*ssbA*	single-stranded DNA binding	DNA recombination/DNA repair	cytoplasm	99	L	x		
***Secondary metabolites biosynthesis, transport and catabolism***										
WP_003151745.1	aldo/keto reductase	*yvgN*	aldo-keto reductase (NADP) activity	small molecule metabolic process	unknown	90	Q	x	x	
WP_003152184.1	DinB family protein	*yuaE*	unknown	unknown	unknown	70	Q	x		
WP_003151318.1	acetolactate decarboxylase	*alsD*	acetolactate decarboxylase activity	acetoin biosynthetic process	unknown	82	Q	x		
WP_004393101.1	VOC family protein	*yycE*	unknown	unknown	unknown	72	Q	x		
WP_197,923,070.1	amino acid adenylation domain-containing protein	*srfAB*	amino acid activation for nonribosomal peptide biosynthetic process/phosphopantetheine binding/	secondary metabolite biosynthetic process	cytoplasm	72	Q	x		
WP_003150879.1	D-alanine–poly(phosphoribitol) ligase subunit DltA	*dltA*	ATP binding/D-alanine [D-alanyl carrier protein] ligase activity	lipoteichoic acid biosynthetic process	cytoplasm	78	Q	x		
WP_015388560.1	NADP-dependent oxidoreductase	*yfmJ*	15-oxoprostaglandin 13-oxidase activity	Aromatic hydrocarbons catabolism/Detoxification	unknown	87	Q	x		
WP_015388106.1	tautomerase family protein	*yolI*	isomerase activity	unknown	unknown	74	Q	x		
***Signal transduction mechanisms***										
WP_003154064.1	RNA chaperone Hfq	*hfq*	RNA binding	translation	cytoplasm	98	T	x	x	x
WP_015388734.1	TerD family protein	*yceC*	unknown	stress response	cytoplasm	82	T	x	x	
WP_003156648.1	TerD family protein	*yceD*	Stress response	Unknown	Unknown	93	T	x	x	
WP_003151456.1	HPr(Ser) kinase/phosphatase	*hprK*	protein serine/threonine kinase activity	regulation of carbohydrate metabolic process	cytoplasm	96	T	x		
WP_004264734.1	cyclic di-AMP receptor DarA	*darA*	unknown	unknown	cytoplasm	97	T	x		
WP_015388712.1	cystine ABC transporter substrate-binding lipoprotein TcyA	*tcyA*	unknown	amino acid transport	plasma membrane	84	TE	x		
WP_003155112.1	YajQ family cyclic di-GMP-binding protein	*yitK*	nucleotide binding	unknown	cytoplasm	88	T	x		
WP_003153343.1	anti-sigma F factor antagonist	*spoIIAA*	anti-sigma factor antagonist activity	sporulation	Unknown	92	T	x		
WP_003152237.1	S-ribosylhomocysteine lyase	*luxS*	S-ribosylhomocysteine lyase activity	quorum sensing	Unknown	93	T	x		
WP_003156645.1	TerD family protein	*yceE*	unknown	unknown	Unknown	95	T	x		
WP_003155391.1	serine/threonine protein kinase PrkA	*prkA*	protein kinase activity	unknown	Unknown	97	T	x		
WP_003154502.1	translational GTPase TypA	*bipA*	GTPase activity	ribosome biogenesis	cytoplasm	97	T	x		
***Transcription***										
WP_015387570.1	DNA-directed RNA polymerase subunit delta	*rpoE*	Nucleotidyltransferase	transcription	cytoplasm	84	K	x		
WP_003152998.1	RNA polymerase sigma factor RpoD	*sigA*	sigma factor activity/DNA binding	transcription regulation	cytoplasm	98	K	x		
WP_003156441.1	DNA-directed RNA polymerase subunit beta'	*rpoC*	DNA-directed 5′−3′ RNA polymerase activity	DNA-templated transcription/response to antibiotic	cytoplasm	98	K	x		
WP_004264660.1	septation regulator SpoVG	*spoVG*	unknown	cell cycle/cell division/cell shape/peptidoglycan synthesis	unknown	96	KD	x		
WP_003152766.1	transcription elongation factor GreA	*greA*	RNA polymerase binding/DNA binding	DNA-templated transcription elongation	cytoplasm	95	K	x		
WP_003154195.1	transcription termination factor NusA	*nusA*	DNA-binding transcription factor activity	DNA-templated transcription termination	cytoplasm	95	K	x		
WP_003156440.1	DNA-directed RNA polymerase subunit beta	*rpoB*	DNA-directed 5′−3′ RNA polymerase activity	DNA-templated transcription/response to antibiotic	cytoplasm	98	K	x		
WP_003154263.1	GTP-sensing pleiotropic transcriptional regulator CodY	*codY*	DNA-binding transcription repressor activity	regulation of DNA-templated transcription	cytoplasm	99	K	x		
WP_003156549.1	DNA-directed RNA polymerase subunit alpha	*rpoA*	DNA-directed 5′−3′ RNA polymerase activity	DNA-templated transcription	cytoplasm	99	K	x		
WP_003151236.1	sporulation transcriptional regulator SpoIIID	*spoIIID*	DNA-binding transcription factor activity	sporulation/ transcription regulation	unknown	100	K	x		
WP_003153177.1	sporulation transcription factor Spo0A	*spo0A*	phosphorelay response regulator activity/ transcription cis-regulatory region binding/calcium ion binding	single-species surface biofilm formation/positive regulation of sporulation resulting in formation of a cellular spore/positive regulation of DNA-templated transcription/Sporulation	cytoplasm	96	KT	x		
WP_003219701.1	two-component system response regulator DegU	*Stat*	DNA-binding	phosphorelay signal transduction system/positive regulation of DNA-templated transcription	cytoplasm	100	KT	x		
WP_169,510,469.1	transition state genes transcriptional regulator AbrB	*abrB*	DNA-binding	sporulation/negative regulation of DNA-templated transcription	unknown	97	KV	x		
***Translation, ribosomal structure and biogenesis***										
WP_003156451.1	elongation factor Tu	*tuf*	GTPase activity	translation	cytoplasm	98	J	x		x
WP_014304716.1	YheC/YheD family protein	*yheC*	ATP binding	sporulation	peripheral membrane protein /spore coat	74	JEHQ	x		
WP_003154288.1	50S ribosomal protein L19	*rplS*	structural constituent of ribosome	translation	cytoplasm	91	J	x	x	x
WP_004264654.1	50S ribosomal protein L25/general stress protein Ctc	*ctc*	5S rRNA binding	translation	cytoplasm	71	J	x	x	
WP_003156433.1	50S ribosomal protein L10	*rplJ*	structural constituent of ribosome	translation	cytoplasm	95	J	x		x
WP_003154296.1	30S ribosomal protein S16	*RPSP*	structural constituent of ribosome	translation	cytoplasm	98	J	x	x	
WP_004393063.1	50S ribosomal protein L9	*rplI*	structural constituent of ribosome/ ARN binding	translation	cytoplasm	91	J	x		x
WP_007410399.1	elongation factor G	*fusA*	translation elongation factor activit	protein biosynthesis	cytoplasm	98	J	x		
WP_003152495.1	50S ribosomal protein L20	*rplT*	Binds directly to 23S ribosomal RNA and is necessary for the *in vitro* assembly process of the 50S ribosomal subunit. It is not involved in the protein synthesizing functions of that subunit (By similarity).	translation	cytoplasm	99	J	x		x
WP_003156436.1	50S ribosomal protein L7/L12	*rplL*	Forms part of the ribosomal stalk which helps the ribosome interact with GTP-bound translation factors. Is thus essential for accurate translation.	Translation	cytoplasm	100	J	x		x
WP_003156464.1	30S ribosomal protein S10	*rpsJ*	large ribosomal subunit rRNA binding	translation	cytoplasm	100	J	x		x
WP_015387878.1	phenylalanine–tRNA ligase subunit beta	*pheT*	Aminoacyl-tRNA synthetase	phenylalanyl-tRNA aminoacylation	unknown	88	J	x		
WP_015387901.1	aspartate–tRNA ligase	*aspS*	aspartate-tRNA ligase activity	protein biosynthesis	cytoplasm	89	J	x		
WP_003152480.1	threonine–tRNA ligase	*thrS*	threonine-tRNA ligase activity	threonyl-tRNA aminoacylation	cytoplasm	93	J	x		
WP_015388055.1	23S rRNA pseudouridine(2605) synthase RluB	*rluB*	23S rRNA pseudouridine(2605) synthase activity	maturation of LSU-rRNA from tetracistronic rRNA transcript (SSU-rRNA, LSU-rRNA, 4.5S-rRNA, 5S-rRNA)	unknown	94	J	x		
WP_003154212.1	ribosome recycling factor	*frr*	ribosomal large subunit binding	translation	cytoplasm	94	J	x		
WP_003154214.1	translation elongation factor Ts	*tsf*	translation elongation factor activity	translational elongation	cytoplasm	94	J	x		
WP_015388789.1	methionine–tRNA ligase	*metG*	methionine-tRNA ligase activity	methionyl-tRNA aminoacylation	cytoplasm	94	J	x		
WP_071543361.1	translation initiation factor IF-3	*infC*	ribosome binding	protein biosynthesis	cytoplasm	94	J	x		
WP_003153515.1	asparagine–tRNA ligase	*asnS*	asparagine-tRNA ligase activity	protein biosynthesis	cytoplasm	95	J	x		
WP_015388783.1	glutamate–tRNA ligase	*gltX*	aminoacyl-tRNA synthetase	protein biosynthesis	cytoplasm	95	J	x		
WP_003156478.1	50S ribosomal protein L16	*rplP*	structural constituent of ribosome	translation/response to antibiotic	cytoplasm	96	J	x		
WP_004392983.1	30S ribosomal protein S18	*rpsR*	structural constituent of ribosome	translation/response to antibiotic	cytoplasm	96	J	x		
WP_007609878.1	Asp-tRNA(Asn)/Glu-tRNA(Gln) amidotransferase subunit GatA	*gatA*	glutaminyl-tRNA synthase (glutamine-hydrolyzing) activity	translation	cytoplasm	96	J	x		
WP_015388311.1	preQ(1) synthase	*queF*	preQ1 synthase activity	queuosine biosynthesis	cytoplasm	96	J	x		
WP_003153123.1	elongation factor P	*efp*	translation elongation factor activity	protein biosynthesis	cytoplasm	97	J	x		
WP_003156499.1	50S ribosomal protein L15	*rplO*	structural constituent of ribosome	translation/response to antibiotic	cytoplasm	97	J	x		
WP_003154182.1	30S ribosomal protein S15	*rpsO*	rRNA binding	translation	cytoplasm	98	J	x		
WP_003156556.1	50S ribosomal protein L13	*rplM*	structural constituent of ribosome	translation/response to antibiotic	cytoplasm	99	J	x		
WP_003156430.1	50S ribosomal protein L11	*rplK*	large ribosomal subunit rRNA binding	translation/response to antibiotic	cytoplasm	100	J	x		
WP_003156467.1	50S ribosomal protein L23		structural constituent of ribosome	translation/response to antibiotic	cytoplasm	100	J	x		
WP_003156472.1	30S ribosomal protein S19	*rpsS*	structural constituent of ribosome	translation/response to antibiotic	cytoplasm	100	J	x		
WP_003154544.1	peptide deformylase	*defB*	peptide deformylase activity	co-translational protein modification	unknown	91	J	x		
WP_003156489.1	30S ribosomal protein S8	*rpsH*	structural constituent of ribosome/ ARN binding	translation	cytoplasm	92	J	x		
WP_003154163.1	ribonuclease J2	*rnjB*	5′−3′ RNA exonuclease activity/RNA endonuclease activity	mRNA processing	cytoplasm	93	J	x		
WP_003152406.1	30S ribosomal protein S4	*rpsD*	structural constituent of ribosome	translation	cytoplasm	95	J	x		
WP_003152493.1	50S ribosomal protein L35	*n bL35*	structural constituent of ribosome	translation	cytoplasm	95	J	x		
WP_003153426.1	30S ribosomal protein S1	*ypfD*	structural constituent of ribosome/ ARN binding	translation	cytoplasm	95	J	x		
WP_003156492.1	50S ribosomal protein L18	*rplR*	structural constituent of ribosome/ ARN binding	translation	cytoplasm	95	J	x		
WP_004392977.1	30S ribosomal protein S6	*rpsF*	structural constituent of ribosome/ ARN binding	translation	cytoplasm	95	J	x		
WP_015388227.1	polyribonucleotide nucleotidyltransferase	*pnp*	3′−5′-RNA exonuclease activity	RNA catabolic process	cytoplasm	95	J	x		
WP_015388229.1	translation initiation factor IF-2	*infB*	translation initiation factor activity	translational initiation	cytoplasm	95	J	x		
WP_003156484.1	30S ribosomal protein S17	*rpsQ*	structural constituent of ribosome/ ARN binding	translation	cytoplasm	96	J	x		
WP_003156490.1	50S ribosomal protein L6	*rplF*	structural constituent of ribosome/ ARN binding	translation	cytoplasm	96	J	x		
WP_003152660.1	50S ribosomal protein L21	*rplU*	structural constituent of ribosome	translation	cytoplasm	97	J	x		
WP_003154550.1	ribonuclease J1	*rnjA*	5′−3′ RNA exonuclease activity/RNA endonuclease activity	mRNA processing	cytoplasm	97	J	x		
WP_003156465.1	50S ribosomal protein L3	*rplC*	structural constituent of ribosome/ ARN binding	translation	cytoplasm	97	J	x		
WP_003156466.1	50S ribosomal protein L4	*rplD*	structural constituent of ribosome/ ARN binding	translation	cytoplasm	97	J	x		
WP_003156476.1	30S ribosomal protein S3	*rpsC*	structural constituent of ribosome/ ARN binding	translation	cytoplasm	98	J	x		
WP_003156487.1	50S ribosomal protein L5	*rplE*	structural constituent of ribosome/ ARN binding	translation	cytoplasm	98	J	x		
WP_003156545.1	30S ribosomal protein S13	*rpsM*	structural constituent of ribosome/ ARN binding	translation	cytoplasm	98	J	x		
WP_003225846.1	30S ribosomal protein S9	*rpsI*	structural constituent of ribosome/ ARN binding	translation	cytoplasm	98	J	x		
WP_003154215.1	30S ribosomal protein S2	*rpsB*	structural constituent of ribosome	translation	cytoplasm	99	J	x		
WP_003156445.1	30S ribosomal protein S12	*rpsL*	structural constituent of ribosome/ ARN binding	translation	cytoplasm	99	J	x		
WP_003156447.1	30S ribosomal protein S7	*rpsG*	structural constituent of ribosome/ ARN binding	translation	cytoplasm	99	J	x		
WP_003156470.1	50S ribosomal protein L2	*rplB*	structural constituent of ribosome/ ARN binding	translation	cytoplasm	99	J	x		
WP_003156485.1	50S ribosomal protein L14	*rplN*	structural constituent of ribosome/ ARN binding	translation	cytoplasm	99	J	x		
WP_003156493.1	30S ribosomal protein S5	*rpsE*	structural constituent of ribosome/ ARN binding	translation	cytoplasm	99	J	x		
WP_003156547.1	30S ribosomal protein S11	*rpsK*	structural constituent of ribosome/ ARN binding	translation	cytoplasm	99	J	x		
WP_003156550.1	50S ribosomal protein L17	*rplQ*	structural constituent of ribosome/ ARN binding	translation	cytoplasm	99	J	x		
WP_003156475.1	50S ribosomal protein L22	*rplV*	structural constituent of ribosome/ ARN binding	translation	cytoplasm	100	J	x		
***Unknown***										
WP_003152178.1	DUF1016 domain-containing protein/biofilm surface layer hydrophobin BslA	*bslA/yuaB*	biofilm formation	unknown/ biofilm formation	extracellular region	74	unknown	x	x	x
WP_015387422.1	DUF1259 domain-containing protein	*ycxD*	unknown/transaminase activity	unknown/alpha-amino acid metabolic process	unknown	27	unknown	x	x	x
WP_003151908.1	hypothetical protein	*mpr*	unknown/serine‑type endopeptidase activity	unknown/proteolysis	unknown/extracellular region	31	unknown	x	x	x
WP_015388185.1	hypothetical protein	*cotC*	unknown	unknown/sporulation	unknown	45	unknown	x	x	x
WP_014306056.1	S8 family serine peptidase	*pnbA*	unknown/cholinesterase activity	unknown	unknown	63	unknown	x	x	x
WP_015388008.1	SipW-dependent-type signal peptide-containing protein/biofilm matrix protein TasA	*tasA*	major protein component of the biofilm extracellular matrix /amyloid fibers that bind cells together in the biofilm/antibacterial/spore coat assembly	sporulation	extracellular region/Forespore intermembrane space	84	unknown	x	x	x
WP_003154111.1	outer spore coat protein CotE	*cotE*	identical protein binding	sporulation	spore coat	96	unknown	x	x	x
WP_014304250.1	DUF4879 domain-containing protein	*yolA*	unknown	unknown	unknown	27	unknown	x	x	
WP_015388007.1	amyloid fiber anchoring/assembly protein TapA	*tapA*	unknown/required for the proper anchoring and polymerization of TasA amyloid fibers at the cell surface	unknown	extracellular region/bacterial biofilm matrix	47	unknown	x	x	
WP_015388281.1	hypothetical protein	*ylaE*	unknown	unknown	unknown	52	unknown	x	x	
WP_003155052.1	peptide-binding protein	*appA*	peptide transmembrane transporter activity	peptide transport/sporulation	periplasmic space	73	unknown	x		x
WP_014305020.1	insulinase family protein	*ymfH*	metallopeptidase activity	proteolysis	unknown	86	unknown	x	x	
WP_015388142.1	IseA DL-endopeptidase inhibitor family protein	*yoeB*	unknown	unknown	unknown	77	unknown	x		x
WP_187,650,984.1	hypothetical protein	*ywmD*	unknown	unknown	unknown	20	unknown	x	x	
WP_014305702.1	hydrolase	*yddQ*	unknown/hydrolase activity	unknown	unknown	31	unknown	x	x	
WP_015387848.1	SGNH/GDSL hydrolase family protein	*yddQ*	unknown/hydrolase activity	unknown	unknown	31	unknown	x	x	
WP_015387689.1	M28 family peptidase	*ywaD*	unknown/peptidase activity	unknown/proteolysis	unknown/extracellular region	48	unknown	x	x	
WP_024084955.1	YdhK family protein	*ydhK*	unknown	unknown	unknown	61	unknown	x	x	
WP_015387670.1	carboxylesterase/lipase family protein	*pnbA*	unknown/cholinesterase activity	unknown	unknown	63	unknown	x	x	
WP_003151188.1	VWA domain-containing protein	*ywmC*	unknown	unknown	unknown	66	unknown	x	x	
WP_003152178.1	DUF1016 domain-containing protein	*bslA/yuaB*	biofilm formation	unknown/Biofilm formation	extracellular region	74	unknown	x	x	
WP_015387776.1	YukJ family protein	*yukJ*	unknown	unknown	unknown	81	unknown	x	x	
WP_015388356.1	phage tail sheath family protein	*xkdK*	unknown	unknown	unknown	86	unknown	x	x	
WP_003152143.1	type 1 glutamine amidotransferase	*yraA*	unknown/peptidase activity	unknown/proteolysis	unknown	38	unknown	x	x	
WP_015388651.1	hypothetical protein	*floT*	unknown	unknown/regulation of cell shape	unknown	28	unknown	x	x	
WP_003154036.1	hypothetical protein	*ssuD*	unknown/alkanesulfonate monooxygenase activity	unknown/alkanesulfonate catabolic process	unknown	30	unknown	x	x	
WP_015387655.1	ribonuclease	*yvaE*	unknown/amino-acid betaine transmembrane transporter activity	unknown/tranport	unknown/plasma membrane	43	unknown	x	x	
WP_003155828.1	DUF1906 domain-containing protein		unknown	unknown	unknown	49	unknown	x	x	
WP_003154023.1	lytic polysaccharide monooxygenase	*ctc*	unknown/Ribonucleoprotein	unknown/translation	unknown/cytoplasm	50	unknown	x	x	
WP_003153543.1	hypothetical protein	*yqgA*	unknown	unknown	unknown/extracellular region	52	unknown	x	x	
WP_003156431.1	50S ribosomal protein L1	*yqgA*	unknown	unknown	unknown/extracellular region	52	unknown	x		x
WP_015387631.1	flagellin	*hag*	structural molecule activity	bacterial-type flagellum	unknown/extracellular region	56	unknown	x		x
WP_004393267.1	hypothetical protein		unknown	unknown	unknown	66	unknown	x	x	
WP_003151740.1	hypothetical protein		unknown	unknown	unknown	81	unknown	x		x
WP_003154135.1	stage V sporulation protein SpoVS	*spoVS*	nucleic acid binding	sporulation //Cell division	unknown	100	unknown	x		x
WP_021734090.1	hypothetical protein	*cotG*	unknown	sporulation	unknown	100	unknown	x		x
WP_015388720.1	GTP-binding protein	*yciC*	GTP binding/hydrolase activity	unknown	unknown	84	unknown	x		x
WP_015388552.1	LPXTG cell wall anchor domain-containing protein	*epr*	serine-type endopeptidase activity	protrolysis	unknown/ extracellular region	22	unknown	x		
WP_015388714.1	aminotransferase class I/II-fold pyridoxal phosphate-dependent enzyme	*aspB*	unknown/L-aspartate:2-oxoglutarate aminotransferase activity	unknown/biosynthetic process	unknown/cytoplasm	25	unknown	x		
WP_015388335.1	glycosyltransferase	*sunS*	unknown	bacteriocin biosynthetic process	unknown	28	unknown	x		
WP_015388287.1	serine hydrolase	*pbpE*	hydrolase	cell wall biogenesis/degradation	unknown	29	unknown	x		
WP_003152402.1	hypothetical protein	*era*	unknown/ribosomal small subunit binding	Unknown/positive regulation of cell division/ribosomal small subunit assembly	unknown/Cytoplasm/Cell membrane	30	unknown	x		
WP_014304383.1	hypothetical protein	*yraI*	unknown	unknown	unknown/spore coat	30	unknown	x		
WP_015387690.1	NAD(P)-dependent oxidoreductase	*ytcB*	dTDP-glucose 4,6-dehydratase activity	UDP-rhamnose biosynthetic process	unknown	31	unknown	x		
WP_015387616.1	glycosyltransferase family 2 protein	*ggaA*	glycosyltransferase	cell wall organization	unknown	33	unknown	x		
WP_003153276.1	cytochrome P450	*yjiB*	unknown/cholest-4-en-3-one 26-monooxygenase activity	unknown/steroid hydroxylase activity	unknown	38	unknown	x		
WP_003155515.1	barstar family protein		unknown	unknown	unknown/cytoplasm	49	unknown	x		
WP_015388034.1	ACP S-malonyltransferase	*pksE*	[acyl-carrier-protein] S-malonyltransferase activity	Unknown/antibiotic biosynthetic process/fatty acid biosynthetic process	unknown/cytoplasm	59	unknown	x		
WP_003152644.1	SPOR domain-containing protein	*spoIIB*	peptidoglycan binding	sporulation	plasma membrane	60	unknown	x		
WP_015388149.1	non-ribosomal peptide synthetase	*ppsD*	phosphopantetheine binding	antibiotic biosynthetic process/amino acid activation for nonribosomal peptide biosynthetic process	unknown	63	unknown	x		
WP_015388212.1	non-ribosomal peptide synthetase		Unknown/fatty acid synthase activity	unknown/fatty acid biosynthetic process	unknown	63	unknown	x		
WP_015388186.1	hypothetical protein	*yvgO*		unknown	unknown	63	unknown	x		
WP_003155509.1	nitroreductase	*yfhC*	Unknown/oxidoreductase activity	unknown	unknown	67	unknown	x		
WP_003155277.1	YheC/YheD family protein	*yheD*	Unknown	unknown/sporulation	Unknown spore coat	67	unknown	x		
WP_003156592.1	RDD family protein	*yckC*	unknown	unknown	unknown/plasma membrane	68	unknown	x		
WP_007409232.1	enoyl-CoA hydratase	*yhaR*	unknown	unknown	unknown/plasma membrane	69	unknown	x		
WP_015387781.1	(*S*)-benzoin forming benzil reductase	*yueD*	sepiapterin reductase activity	tetrahydrobiopterin biosynthetic process	unknown/cytoplasm	69	unknown	x		
WP_003154085.1	sporulation protein	*nucB*	nuclease activity	sporulation	extracellular region	70	unknown	x		
WP_046341317.1	hypothetical protein	*cgeC*	unknown	unknown	unknown	71	unknown	x		
WP_015388097.1	hypothetical protein	*yodI*	unknown	unknown	cell membrane	71	unknown	x		
WP_003152478.1	putative sporulation protein YtxC	*ytxC*	unknown	unknown	unknown	75	unknown	x		
WP_003154479.1	hypothetical protein	*ylbA*	unknown	unknown	unknown	75	unknown	x		
WP_003154632.1	hypothetical protein	*ykwD*	unknown	unknown	unknown	76	unknown	x		
WP_003155695.1	VOC family protein	*yetH*	unknown	unknown	unknown	76	unknown	x		
WP_003156589.1	competence protein ComJ	*nin*	unknown	establishment of competence for transformation	plasma membrane	78	unknown	x		
WP_015387536.1	spore coat protein GerQ	*gerQ*	isopeptide cross-linking via N6-(L-isoglutamyl)-l-lysine	sporulation	spore coat	79	unknown	x		
WP_003153148.1	stage III sporulation protein AG	*spoIIIAG*	unknown	sporulation	plasma membrane	80	unknown	x		
WP_003151446.1	DUF4097 domain-containing protein	*yvlB*	unknown	unknown	plasma membrane	81	unknown	x		
WP_015388178.1	CoA-binding protein	*yneT*	unknown	unknown	unknown	85	unknown	x		
WP_003154178.1	insulinase family protein	*ymxG*	metalloendopeptidase activity	proteolysis	unknown	90	unknown	x		
WP_003152380.1	YtxH domain-containing protein	*ytxH*	unknown	unknown	unknown	92	unknown	x		
WP_004264624.1	VWA domain-containing protein	*yabS*	unknown	unknown	unknown	93	unknown	x		
WP_003152958.1	GatB/YqeY domain-containing protein	*yqeY*	carbon-nitrogen ligase activity, with glutamine as amido-N-donor		unknown	93	unknown	x		
WP_003153984.1	small acid-soluble spore protein P	*sspP*	asexual sporulation	sporulation	spore coat	97	unknown	x		
WP_003218330.1	protein Veg	*veg*	regulation of DNA-templated transcription	sporulation	cytoplasm/spore core	100	unknown	x		
WP_003154969.1	YjcG family protein	*yjcG*	hydrolase activity, acting on ester bonds	unknown	unknown	80	unknown	x		
WP_015387693.1	PIG-L family deacetylase	*bshB1*	unknown/hydrolase activity, acting on carbon-nitrogen (but not peptide) bonds, in linear amides	unknown/bacillithiol biosynthetic process	unknown	26	unknown	x		
WP_003153268.1	SDR family oxidoreductase		unknown	unknown	unknown	37	unknown	x		
WP_003155166.1	YbjQ family protein		unknown	unknown	unknown	37	unknown	x		
WP_014304855.1	pyocin knob domain-containing protein	*xkdV*	unknown	unknown	unknown	37	unknown	x		
WP_015388166.1	non-ribosomal peptide synthetase	*ppsE*	unknown	unknown	unknown	37	unknown	x		
WP_003156618.1	antimicrobial peptide LCI	*yoqM*	unknown	unknown	unknown	38	unknown	x		
WP_015388164.1	non-ribosomal peptide synthetase	*ppsD*	unknown/phosphopantetheine binding	unknown/Antibiotic biosynthesis	unknown/Cytoplasm	40	unknown	x		
WP_015388165.1	non-ribosomal peptide synthetase	*srfAB*	unknown/ligase activity	unknown/Antibiotic biosynthesis	unknown/Cytoplasm	41	unknown	x		
WP_015388199.1	SMP-30/gluconolactonase/LRE family protein	*pksN*	unknown/fatty acid synthase activity	Unknown/fatty acid biosynthetic process	unknown/Cytoplasm	41	unknown	x		
WP_021734242.1	aldo/keto reductase	*yvgN*	unknown/aldo-keto reductase (NADP) activity	unknown/small molecule metabolic process	unknown	43	unknown	x		
WP_015388035.1	SDR family NAD(P)-dependent oxidoreductase		unknown	unknown	unknown	45	unknown	x		
WP_175,415,675.1	spore coat protein	*cotO*	unknown	unknown/sporulation	unknown	50	unknown	x		
WP_014306116.1	pectate lyase	*pelB*	unknown/pectin lyase activity	unknown	unknown/extracellular region	55	unknown	x		
WP_015388499.1	5′-nucleotidase C-terminal domain-containing protein	*yhcR*	unknown/5′-nucleotidase activity	unknown/nucleotide catabolic process	unknown/extracellular region	64	unknown	x		
WP_015387766.1	S9 family peptidase	*yuxL*	unknown/serine‑type endopeptidase activity	unknown/proteolysis	unknown	68	unknown	x		
WP_015387578.1	VWA domain-containing protein	*ywmD*	unknown	unknown	unknown	72	unknown	x		
WP_015388152.1	family 10 glycosylhydrolase	*yngK*	hydrolase activity, acting on glycosyl bonds	metabolic process	unknown	73	unknown	x		
WP_003154450.1	sporulation-specific transcriptional regulator GerR	*ylbO*	DNA-binding	unknown	unknown	79	unknown	x		
WP_014304356.1	aldo/keto reductase family oxidoreductase	*ycsN*	oxidoreductase activity	unknown	cytoplasm	81	unknown	x		
WP_003153362.1	DUF1002 domain-containing protein	*ypuA*	unknown	unknown	plasma membrane	84	unknown	x		
WP_015387548.1	cupin domain-containing protein	*bacB*	intramolecular oxidoreductase activity, transposing *C* = *C* bond/cobalt ion binding	antibiotic biosynthetic process	cytoplasm	84	unknown	x		
WP_015388696.1	pyridoxamine 5′-phosphate oxidase family protein	*ydaG*	unknown	stress response	unknown	86	unknown	x		
WP_003153156.1	Asp23/Gls24 family envelope stress response protein	*yqhY*	unknown	unknown	unknown	91	unknown	x		
WP_003154415.1	YlmC/YmxH family sporulation protein	*ylmC*	unknown	sporulation	unknown	92	unknown	x		
WP_003154717.1	alpha/beta-type small acid-soluble spore protein	*sspD*	double-stranded DNA binding	sporulation	unknown	92	unknown	x		
WP_007408043.1	alpha/beta-type small acid-soluble spore protein	*sspA*	double-stranded DNA binding	DNA topological change/Sporulation	unknown	92	unknown	x		
WP_003152961.1	flotillin-like protein FloA	*floA*	unknown	unknown	plasma membrane	93	unknown	x		
WP_003154908.1	DUF1641 domain-containing protein	*yjlC*	unknown	unknown	unknown	95	unknown	x		
WP_003153449.1	trp RNA-binding attenuation protein MtrB	*mtrB*	DNA-templated transcription termination/RNA binding	negative regulation of translational initiation	unknown	96	unknown	x		
WP_003154837.1	phage tail tube protein	*xkdM*	unknown	unknown	unknown	97	unknown	x		
WP_004264638.1	sporulation protein YabP	*yabP*	unknown	sporulation	membrane/endospore-forming forespore	98	unknown	x		
WP_004264630.1	RNA-binding protein S1	*yabR*	unknown	unknown	unknown	99	unknown	x		

a Accession number.

b Protein name in the NCBI database for *B. amyloliquefaciens*l-17.

c Gene designation.

d Subcellular localization.

e molecular function and ^f^biological process data obtained from the UniProKB database after BLAST sequence similarity searches.

g Identity ( %) in UniProt Protein BLAST: percentage of identical amino acids between the *B. amyloliquefaciens*l-17 proteins sequence and that of *B. subtilis* 168.

h COG category symbole: functional categories according Clusters of Orthologous Genes (COG) database.

i CFLP: proteins extracted on the liquid phase underlying the pellicle.

j LB-fraction:proteins loosly bound to the matrice that were detached by mechanical disruption of the pellicle.

k TB-fraction: proteins tightly bound to the matrice that were chemically extracted of the pellicle. In green, the identified proteins in l-17 strain having low % identity (<70 %) with that of *B. subtilis* 168.

#### 3.3.2 Comparison of pellicle proteins and CFLP associated proteins identified in *B. amyloliquefaciens*l-17

A total of 118 proteins were concomitantly identified in the pellicle and CFLP (Pell-CFLP proteins). Most of them (53 %) are from the LB-fraction. This is not surprising since the proteins of this fraction are weakly linked to the pellicle matrix, facilitating their transition to CFLP. However, 31 % of the Pell-CFLP proteins were found in the TB-fraction and 16 % are found both in the LB, TB and in CFLP samples (Table 2). These results support the possible exchange relationships that could exist between planktonic *B. amyloliquefaciens* l-17, and their counterparts engaged in pellicle lifestyle. Indeed, this dynamic relationship could involve the globality of matrix proteins and would not be limit to those of the EPSs weakly bound layer.

We focused then on Pell-CFLP proteins that are most abundant in the pellicle (TB and /or LB fractions) and are also prevalent in CFLP (i.e. Tas A and BslA proteins, flagellin or Bpr serine peptidase) (Fig. 4). Fig. 4b shows a comparison of the abundance of these proteins in LB, TB and CFLP samples. Tas A is over presented in the pellicle and seems to be tightly bound to the matrix. Its identification in the CFLP is unsurprising. The role of TasA in the structure of the *B. subtilis* biofilm is well documented, but it is also produced at the beginning of spore formation, when the cells enter stationary phase, and is secreted into the medium as well as deposited in the spore (Stöver and Driks, 1999). BslA appears to follow the same scenario. This secreted protein forms surface hydrophobic layers around the biofilm of *Bacillus* species and renders it water-repellent (Morris et al., 2017). The Bpr extracellular protease is also more abundant in the pellicle than the CFLP reflecting a particular importance of Bpr in the pellicle matrix. Flagellin is over presented both in the LB-fraction and CFLP. This supports the hypothesis of the flagellin importance in the pellicle either to provide mobility to cells that leaves biofilm or to specialize the subpopulation of stealth swimmers giving the pellicle elasticity (Section 3.1).

#### 3.3.3 Subcellular localization and biological function of CFLP associated proteins

The subcellular localization and functional categories of each protein are reported in Table 2 and Fig. 4a shows the distributions of CFLP proteins based on these data.

**Fig. 5:**
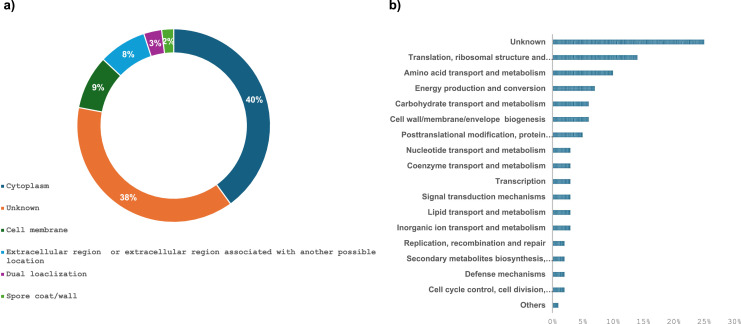
(a) Subcellular localization and (b) functional categories of the cell free liquid phase associated proteins identified in *B. amyloliquefaciens*l-17. The proteins with dual localization can be associated to membrane/cytoplasm, cell membrane/spore or cytoplasm/spore. The Cell membrane localization groups together plasma membrane, periplasmic space and bacterial flagellum. The category “Others" groups together functional categories with <3 proteins (i.e. mobilome: prophages, transposons; Intracellular trafficking, secretion, and vesicular transport).

The investigation of cellular location showed that 8 % of CFLP proteins were predicted as strictly extracellular or extracellular with another possible location (Fig. 5a). Of the 10 most CFLP abundant proteins (Fig. 4a), 8 fit into this scenario (Table 2). We noted that this category includes 10 proteins (27 %) identified only in CFLP and were not found in pellicle (Table 2). These are mainly enzymes, which would be produced and released into the extracellular environment by planktonic bacteria. Half of the proteins are originally from the cytoplasm, the periplasmic space or the plasma membrane. This indicates significant cell lysis given that the culture is 72 h old, and the environment is no longer very favorable to bacterial growth. The residence of predicted cytoplasmic and cell membrane proteins in the extracellular proteome has also been reported and discussed in *B. subtilis* by Tjalsma et al. (2004). In addition to the obvious hypothesis of cell lysis, these authors support that these proteins would leave the cellular compartments *via* flagellar export machinery, the holin systems, or other unidentified export systems. We also found a few proteins (2 %) related to the spore in CFLP but most of them were also identified in the pellicle. Finelly, the subcellular localization of 164 proteins (38 %) remains unknown. Among these proteins, 105 (24 % of CFLP identified proteins) have not described localization in the databases and 59 proteins (14 % of CFLP identified proteins) present <70 % sequence identity with their homologous in *B. subtilis* 168 (Table 2). Because of this low homology, we cannot transpose the information available on the proteins of *B. subtilis* to that of *B. amyloliquefaciens*. We have therefore included these proteins in the unknown category.

All the identified 429 CFLP proteins were classified in clusters of orthologous genes (COGs) functional categories (Fig. 5b). CFLP identified proteins are involved in various physiological processes. We then focused on the 310 proteins only identified in the CFLP (Table 2), admitting that the remainder of proteins that simultaneously found in CFLP and pellicle have already been discussed in the previous paragraphs. We noted that the molecular function and biological process of a large proportion (23 %) of proteins are not known. We identified 5 non-ribosomal peptide synthases (WP_015388164.1, WP_015388149.1, WP_015388166.1, WP_015388212.1, WP_015388165.1) which reflect the presence of non-ribosomally synthesized antimicrobial peptides in CFLP (Table 2). In addition, we identified the amino acid adenylation domain-containing protein (WP_197,923,070.1) homologous of surfactin synthase A of *B. subtilis* 168 (72 % sequence identity). This suggests the presence of surfactin, a well-studied biosurfactant with antifungal properties, produced by various *Bacillus* species such as *B. amyloliquefaciens, B. subtilis*, and *B. pumilus* (Jacques, 2011; Ranjan et al., 2023)*.* This may also be supported by the presence of the peptide ABC transporter substrate-binding protein (WP_015388449.1), the *oppA* gent product, among the most abundant CFLP identified proteins (Fig. 4a). Indeed, OppA plays a key role in surfactin synthesis regulation in *B. subtilis* (Wang et al., 2019). A targeted mass spectrometry analysis, aimed at identifying antimicrobial peptides in CFLP, would be necessary to characterize these bioactive molecules. Moreover, we identified a panel of enzymes in the CFLP in which the trifunctional nucleotide phosphoesterase YfkN, glucuronoxylanase xynC and the catalase KatA were over presented (Fig. 4a). These enzymes were also characterized in the cell free supernatant of the *B. subtilis* planktonic (Chambert et al., 2003; Naclerio et al., 1995; St. John et al., 2006). Other enzymes including proteases, lipases, aminotransferases, peroxidases and phytase were also characterized in CFLP (Table 2). This result is not surprising since *B. amyloliquefaciens* and other *Bacillus* species have long been recognized to produce commercial enzymes having biotechnological applications including textile applications, feed industry, food industry, and organic synthesis (Farhat-Khemakhem et al., 2018; Ngalimat et al., 2021; Rizwanuddin et al., 2023).

Based on our findings, we can conclude that the liquid phase underlying the pellicle of l-17 strain should not be overlooked. It requires particular interest since (i) it seems to be the site of collaboration and regulation between the mobile and sessile bacterial subpopulations and (ii) it could be a source of commercial enzymes and natural antimicrobial agents applicable in various fields.

## 4 Conclusion

The present study provides the first global picture of proteins both in the pellicle matrix and in the liquid phase of *B. amyloliquefaciens* l-17 occurred by high-throughput proteomic approach. For the pellicle matrix proteins characterization, the combination of physical (ULTRA-TURRAX grinding) and chemical (NaOH treatment) technique es of EPSs extraction followed by the MS analysis allowed to establish a list of 131 pellicle associated proteins which can loosely or tightly bound to the matrix. The identified proteins are involved in various physiological processes. The main identified proteins in the pellicle of l-17 strain are TasA, TapA, and BslA whose roles in the biofilm architecture and proprieties are well described in *B. subtills*. The literature available data and the interaction network highlighted that these proteins deserve to be genetically and biochemically explored to provide more explanations and information on their role in the physiology of the air-liquid interface biofilm in l-17 strain.

The identification of the flagellin and the related-sporulation proteins suggests a cellular specialization and a repartition of labor within the pellicle. Further microscopy studies are necessary to explore this hypothesis. Since the protein composition of the matrix is continually evolving, it would be interesting to perform proteomic analysis at different stages of pellicle life. This should be completed by the proteomic characterization of cellular proteins extracted from bacteria associated pellicle. Together, these investigations would allow us to highlight important molecular players in the formation, maintenance and regulation of the pellicle.

Proteomic characterization of the liquid phase underlying the pellicle showed that this fraction is rich in proteins since 423 proteins were identified. Among them, some have also been identified in the matrix of the pellicle. This strengthens the idea of dynamic exchanges between the sessile and planktonic populations. Liquid phase specific proteins include enzymes involved in the biosynthesis process of non-ribosomal peptides. Studies, such as targeted MS, seem to be necessary to characterize these bioactive agents. A variety of commercial enzymes are also characterized. This finding can promote the application of liquid phase in biocontrol, and in different industrial and agricultural fields.

Ultimately, this study constitutes the foundation for further biological studies to (i) draw conclusions regarding the pellicle formation and regulation (ii) understand its spatial and the architectural organization (iii) elucidate social interactions and the division of labor in the microbial community (iv) decipher the molecular interaction network between the sessile and planktonic population (v) highlight the application of the cell-free liquid phase in industry and agriculture.

## CRediT authorship contribution statement

**Tassadit Ouidir:** Writing – original draft, Methodology, Data curation, Conceptualization. **Julie Hardouin:** Investigation, Data curation, Formal analysis. **Claire-Emmanuelle Marcato-Romain:** Writing – review & editing. **Elisabeth Girbal-Neuhauser:** Visualization, Writing – review & editing, Investigation. **Yassine Nait Chabane:** Conceptualization, Methodology, Writing – review & editing, Supervision, Project administration.

## Declaration of competing interest

The authors declare that they have no known competing financial interests or personal relationships that could have appeared to influence the work reported in this paper.

## Data Availability

The authors are unable or have chosen not to specify which data has been used.

## Appendix Supplementary materials

Image, application 1

Image, application 2
