# Supplementary material for: Large scale identification of pellicle and cell-free liquid phase associated proteins in Bacillus amyloliquefaciens L-17
Source: Curr Res Microb Sci. 2025 Apr 8;8:100387. doi: 10.1016/j.crmicr.2025.100387 (PMC12020853; doi:10.1016/j.crmicr.2025.100387)
Supplement: Supplementary file 1 [file mmc1.docx]

**Appendix A. Supporting information.**

**Table SI:** Details of proteins identification in each biological replicate of LB, TB and CFLP fractions, including PSM (Peptide Spectral Matching, indicator of protein abundance).
